# Interspecies cross-feeding orchestrates carbon degradation in the rumen ecosystem

**DOI:** 10.1038/s41564-018-0225-4

**Published:** 2018-10-24

**Authors:** Lindsey M. Solden, Adrian E. Naas, Simon Roux, Rebecca A. Daly, William B. Collins, Carrie D. Nicora, Sam O. Purvine, David W. Hoyt, Julia Schückel, Bodil Jørgensen, William Willats, Donald E. Spalinger, Jeffrey L. Firkins, Mary S. Lipton, Matthew B. Sullivan, Phillip B. Pope, Kelly C. Wrighton

**Affiliations:** 1 Department of Microbiology, The Ohio State University, Columbus, OH USA; 2 Faculty of Chemistry, Biotechnology and Food Science, Norwegian University of Life Sciences, Aas, Norway; 3 Alaska Department of Fish and Game, Division of Wildlife Conservation, Palmer, AK USA; 4 Pacific Northwest National Laboratory, Richland, WA USA; 5 Department of Plant and Environmental Sciences, University of Copenhagen, Copenhagen, Denmark; 6 School of Natural and Environmental Sciences, Newcastle University, Newcastle upon Tyne, UK; 7 Department of Biology, University of Alaska Anchorage, Anchorage, AK USA; 8 Department of Animal Sciences, The Ohio State University, Columbus, OH USA; 9 Department of Civil, Environmental and Geodetic Engineering, The Ohio State University, Columbus, OH USA

**Keywords:** Metagenomics, Proteomics, Microbiome

## Abstract

Because of their agricultural value, there is a great body of research dedicated to understanding the microorganisms responsible for rumen carbon degradation. However, we lack a holistic view of the microbial food web responsible for carbon processing in this ecosystem. Here, we sampled rumen-fistulated moose, allowing access to rumen microbial communities actively degrading woody plant biomass in real time. We resolved 1,193 viral contigs and 77 unique, near-complete microbial metagenome-assembled genomes, many of which lacked previous metabolic insights. Plant-derived metabolites were measured with NMR and carbohydrate microarrays to quantify the carbon nutrient landscape. Network analyses directly linked measured metabolites to expressed proteins from these unique metagenome-assembled genomes, revealing a genome-resolved three-tiered carbohydrate-fuelled trophic system. This provided a glimpse into microbial specialization into functional guilds defined by specific metabolites. To validate our proteomic inferences, the catalytic activity of a polysaccharide utilization locus from a highly connected metabolic hub genome was confirmed using heterologous gene expression. Viral detected proteins and linkages to microbial hosts demonstrated that phage are active controllers of rumen ecosystem function. Our findings elucidate the microbial and viral members, as well as their metabolic interdependencies, that support in situ carbon degradation in the rumen ecosystem.

## Abstract (web summary)

A combination of proteomics, metagenome-assembled genomes and heterologous gene expression experiments reveals a trophic system for carbon utilization in the moose rumen microbiome and provides insights into phage dynamics in this ecosystem.

## Main

Ruminant animals harness energy from plant material using the power of interacting microorganisms, which break down plant carbon into short-chain fatty acids (SCFAs)^1^. Previous single-gene studies of the rumen microbiome have revealed that the most abundant and prevalent rumen microorganisms are taxonomically unassigned; yet, these taxa are conserved across many species of ruminants^2–4^. Metagenomic investigations have recovered thousands of genomes from uncultivated rumen microbial lineages. These genomes have defined taxonomic groups^5,6^, providing a metabolic blueprint for some of these prevalent and uncultivated taxa^2,6,7^, and a profile of the carbohydrate-active enzymes that are used to break down carbon^6,8,9^. The impact of viruses in the rumen is also beginning to be understood. Recent studies have demonstrated that rumen viral populations are taxonomically unassigned^10,11^, encode auxiliary metabolic genes^12^, change in abundance with dietary amendments^12^ and can lyse metabolically important ruminant bacteria^13^. Despite these great intellectual and technical advances, we still lack a systems-level understanding of how bacterial and viral metabolic potential is manifested in the rumen to affect carbohydrate processing.

To address this knowledge gap, we leveraged our previous sampling of 24 time-series rumen fluid samples collected from Alaskan moose foraging on a seasonal lignocellulose diet spanning the spring, summer and winter months^14^. Compared to previous ruminant studies, which used hunter-killed animals, rumen-fistulated moose provided unparalleled access to rumen microorganisms, expressed proteins and metabolomes from live animals as they consume and digest natural forage. We previously reported that moose consuming a winter diet high in recalcitrant woody biomass (for example, twigs and bark) had conserved chemistry and microbiology across multiple moose and time periods^14^. Rumen fluid from winter diets, relative to spring and summer diets, had significant increases in the levels of lignin and hemicelullose, which enriched for microbial communities composed of genomically unsampled and enigmatic Bacteroidetes members^14^. Many of these Bacteroidetes and other enriched taxa were described as core members conserved across 35 different species of ruminant animals^2^, suggesting that they may play important roles in the degradation of recalcitrant carbon and provide benefits to host metabolism.

Here, we resolve the physiological roles, substrate preferences and metabolic exchanges for these uncultivated taxa enriched in a high lignocellulosic environment. We deeply sequenced four metagenomes from a size-fractionated rumen fluid sample representative of the winter diet^14^. This approach recovered quality viral and bacterial genomes from low-abundance members, creating a genome database to annotate metaproteome data and link expression data to metabolite sources and sinks. The integration of these data enabled us to (1) phylogenetically resolve and define previously unknown taxonomic clades, (2) predict substrates from polysaccharide utilization loci (PULs) and (3) elucidate viral–host interactions. This study deciphers the trophic structure and metabolic handoffs underpinning a lignocellulosic ecosystem, with findings relevant to agriculture, human health and biofuel production.

## Results and discussion

### Taxonomic and metabolic classification of rumen metagenome-assembled genomes

To broadly sample plant-associated and planktonic microorganisms, we obtained 53.8 Gbps of Illumina HiSeq sequencing data from one size-fractionated rumen fluid sample. This included separate metagenomes for microorganisms associated with (1) plant particulate matter, those retained on a (2) 0.8-μm filter and (3) 0.2-μm filter, as well as (4) viral and small cells that pass through a 0.2-μm filter (Supplementary Fig. 1). These four metagenomes total at least twice as many reads per sample than previous rumen metagenome studies^6,7^, allowing us to reconstruct 356 metagenome-assembled genomes (MAGs). Because our primary goal was to profile the expressed genomic potential that contributed to the rumen carbohydrate food web, we focused on 77 unique bacterial and archaeal MAGs that were near complete (>75%) and 810 unique viral contigs (>10,000 bp). A majority (71%) of these MAGs belonged to uncultivated lineages that lacked any previous metabolic or phylogenetic insights (Supplementary Dataset 1, Supplementary Table 1). Based on the recently proposed Genomic Standards Consortium standards^15^, all genomes are at least medium quality, with 11 categorized as high quality (Supplementary Dataset 1, Supplementary Tables 1 and 2). These genomes sampled bacterial members that we demonstrated by 16S ribosomal RNA amplicon sequencing to be enriched exclusively on the winter diet, relative to the summer or spring diets^14^ (Supplementary Fig. 2). This selective increase in 16S rRNA gene copy number may hint at functional responses to increased woody plant biomass in this winter dietary treatment.

**Fig. 1:**
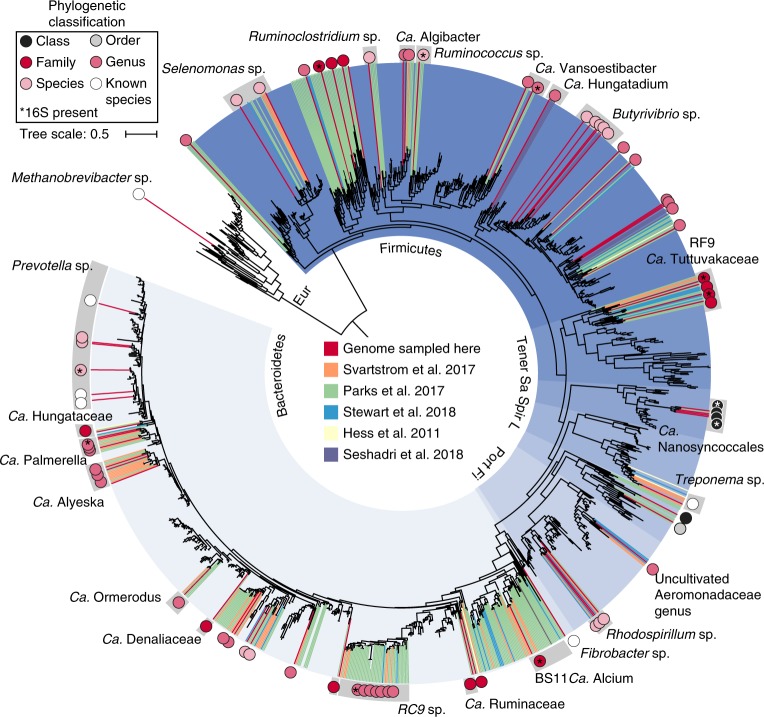
Phylogeny and genomic sampling of 77 rumen MAGs. Maximum likelihood tree of the ribosomal protein rpsC (S3) with reference genomes (3,140), genomes from other rumen MAG studies (345) and genomes recovered here (68). Branches are marked with coloured lines by the rumen data set where the genome originated (centre legend). Coloured circles on the outside of the tree highlight the highest-level taxonomic classification that genomes recovered in this study represent the sampling of (top left legend). The asterisks indicate MAGs containing a partial 16S rRNA gene sequence (>300 bp). The tree is rooted by Euryarchaeota (Eur). Phylum-level groups are outlined in shades of blue and are labelled on the inside circle (Tener, Tenericutes; Fi, Fibrobacteres; Sa, Saccharibacteria (TM7); Spir, Spirochaetes; L, Lentisphaera; Prot, Proteobacteria). Named groups have grey shading behind the circles, including genomes belonging to known genera. The full tree in Newick format is provided in Supplementary Dataset 11. Note, the red lines are missing for the RC9 gut group genomes because they did not contain rpsC proteins; however, the placement of these genomes was confirmed by concatenated ribosomal protein analyses (Fig. 3).

MAGs were taxonomically assigned based on the congruence of multiple phylogenetic trees (Supplementary that can be found in newick format in Datasets 2–26). To aid in the resolution of undefined taxonomic groups, we also recruited 345 genomes from published metagenomics data sets (UBA genomes, the Hungate 1000 project and other rumen MAGs)^5–7,9,16^. Our comprehensive phylogenetic analyses, which included all rumen genomes sampled to date, allowed us to resolve previously unknown classes (two), orders (one), families (four) and genera (nine) across six phyla (Fig. 1, Supplementary Fig. 3 and Supplementary Dataset 1 Supplementary Tables 1 and 3). Three of these lineages are composed entirely of genomes recovered from rumen data sets, suggesting a lineage–ecosystem relationship for these organisms (Bacteroidetes *Candidatus* Ruminaceae and *Candidatus* Hungataceae, and Firmicutes *Candidatus* Hungatadium). A detailed taxonomic, metabolic and naming description for each of our MAGs is provided (Supplementary Discussion). The remaining 29% of our genomes belong to genera with existing cultivated representatives: *Prevotella* (six), *Rhodospirillum* (three), *Ruminococcus* (one), *Ruminoclostridium* (one), *Selenomonas* (two), *Butyrivibrio* (five), *Methanobrevibacter* (one), *Fibrobacter* (one) and *Treponema* (one). Using a combination of genomes from our study, as well as those previously unassigned from other rumen metagenome studies, we provide the metabolic context for 180 genomes belonging to 12 previously undescribed lineages (Fig. 1, Supplementary Fig. 3, Supplementary Dataset 1, Supplementary Table 3 and Supplementary Discussion).

**Fig. 2:**
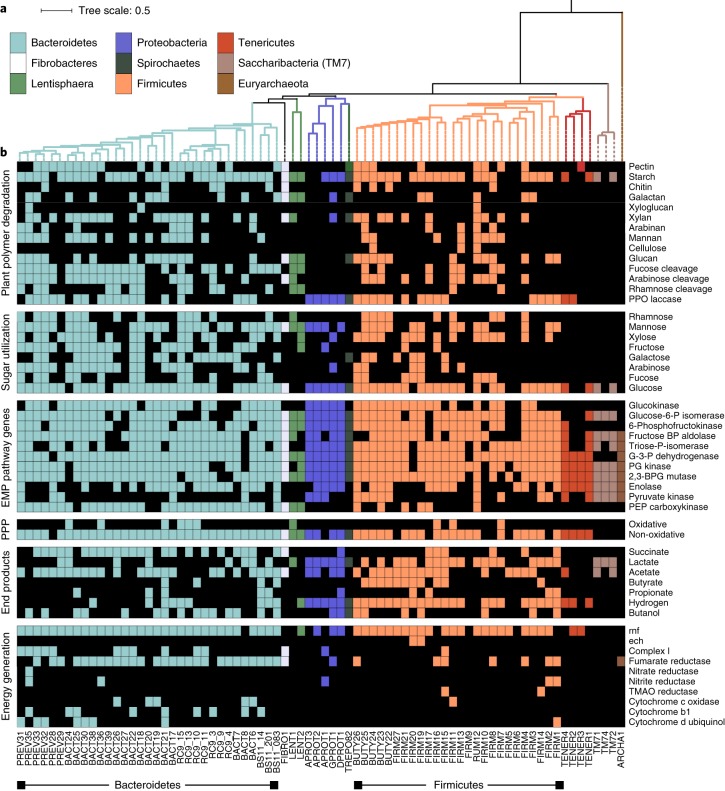
Metabolic reconstruction of all 77 unique near-complete genomes in this study. **a**, Maximum likelihood tree of 16 concatenated ribosomal proteins from all 77 MAGs recovered in this study. Branches are coloured by phyla. Full tree in newick format is provided in Supplementary Dataset 12. **b**, Heat map showing the presence of genes or pathways (listed on the right) found in each MAG (bottom). The presence of a gene or pathway is denoted by a box, coloured by taxonomic assignment. Genes or pathways that were not detected in that MAG are represented with a black box. For pathway completion, 60% of the pathway needed to be present. The functional category is denoted on the left-hand side. BP, bisphosphate; BPG, bisphosphoglycerate; G-3-P, glyceraldehyde-3-phosphate; P, phosphate; PG, phosphoglycerate; PPO, polyphenol oxidase; PPP, pentose phosphate pathway; TMAO, trimethylamine-*N*-oxide; PEP, phosphoenolpyruvate; rnf, Ferredoxin:NAD^+^-oxidoreductase; ech, ech-type hydrogenase.

Metabolic reconstruction of our 77 unique MAGs revealed that the capacity to use plant polymers and sugars was predominant across phyla, with starch and glucose the most well-represented substrates (Fig. 2, Supplementary Dataset 1, Supplementary Table 4). Acetate production was widely encoded, whereas the production of other SCFAs was mainly constrained to the Firmicutes and Bacteroidetes. Similar to MAGs recovered from the human gut^17^, our genomes encode the capacity for respiratory metabolism using fumarate, nitrate, nitrite and trimethylamine-*N*-oxide as electron acceptors (Fig. 2 and Supplementary Discussion). Although these MAGs have broader metabolic capabilities than fermentation alone, these proteins were not detected in metaproteomics. Proteins for polymer degradation, sugar utilization and SCFA production were all highly detected in metaproteomic data from many different organisms. This functional redundancy, consistent with other studies in human and animal gastrointestinal tracts^18–20^, may sustain the production of host beneficial metabolites (for example, SCFAs) under different dietary regimes^18–20^.

### PULs are critical for rumen carbon degradation

**Fig. 3:**
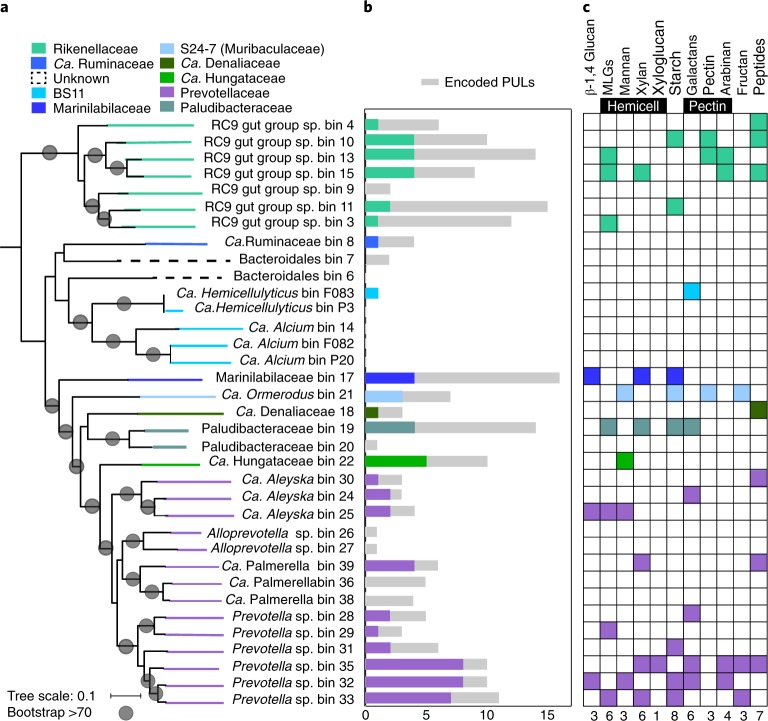
Detection and expression of PULs across known and previously undescribed Bacteroidales members. **a**, Maximum likelihood phylogenetic tree of 16 concatenated ribosomal proteins from all 32 Bacteroidetes MAGs recovered here. These genomes span at least ten families within the Bacteroidales order. Lines connecting the tree to the genome name are coloured by taxonomic family assignment. Circles represent nodes with bootstrap support greater than 70, out of 100 bootstraps. Tree scale defines branch length. The full tree in Newick format is provided in Supplementary Dataset 13. **b**, The number of PULs encoded (grey) and expressed (coloured) from genomes. **c**, Expressed PULs organized by substrate. The coloured boxes indicate that at least one gene within the PUL was detected in proteomics, with two or more unique peptides. Full descriptions of PULs and detected proteins are given in Supplementary Dataset 1, Supplementary Table 5. MLGs, mixed-linked glucans.

Our metaproteomics data demonstrate the important role of Bacteroidetes in rumen carbon degradation, with the Bacteroidetes genomes encoding 91% of the 84 total glycoside hydrolase proteins detected. These glycoside hydrolases belong to genomes from established taxonomies and previously unknown families and genera (Fig. 3a). Consistent with previous reports from cultivated representatives, these Bacteroidetes glycoside hydrolases are located within PULs^21–23^. PULs were defined on assembled contigs as gene clusters containing SusCD-like proteins surrounded by enzymes (including glycoside hydrolase genes) that enable the bacterium to recognize, import and degrade polymeric carbohydrates^22,24^. Similar to previous findings in assembled metagenomes of moose rumen fluid enrichments^25^, PULs in our metagenomes contain many glycoside hydrolase family 43 (GH43) and GH13, with 32 and 15 of our PULs containing these CAZymes, respectively. In addition to PULs, and consistent with previous rumen metagenome studies^6^, we recovered cellulosome-affiliated genes encoded exclusively in Firmicutes genomes. Here, we show, on a woody biomass diet, that Firmicutes cellulosome-derived glycoside hydrolases were not well represented in the metaproteome, despite using protein extraction methods previously recommended for Gram-positive organisms in the human gut^26^.

To specifically link these PULs to detected plant polymers in the rumen, we compared SusCD-like protein pairs to PULs identified and biochemically characterized in PULDB^27^. Similar to previous publications, we found many (36) SusCD-like proteins without CAZymes^6,7,25^. We identified 198 PULs with CAZymes and peptidases, 35% of which had at least one of the co-localized genes (SusCD like or glycoside hydrolase) detected in proteomics (Fig. 3b, Supplementary Dataset 1 Supplementary Table 5). More than half of the expressed PULs were encoded in RC9 gut group and *Prevotella* MAGs (Fig. 3b). From three *Prevotella* genomes (PREV35, PREV32 and PREV33), closely related to *P.* *ruminicola*, we detected peptides from many different PUL types, probably enabling the degradation of various hemicellulose and pectin polymers (xylan, mannan, arabinan and galactan). For each of the other *Prevotella* genomes, closely related to *P.* *buccae*, we only detected peptides from one PUL, with each genome utilizing a different substrate. The proteomics findings show that niche differentiation occurs at the strain level, as the *Prevotella* genomes did not have overlapping polymer degradation profiles. This flexible foraging behaviour has been seen with other members of the Bacteroidetes in the human gut^28^. However, here, the functional differentiation has been shown using MAGs from uncultivated microorganisms.

### Biochemical characterization validates metaproteomic inferences of PULs

**Fig. 4:**
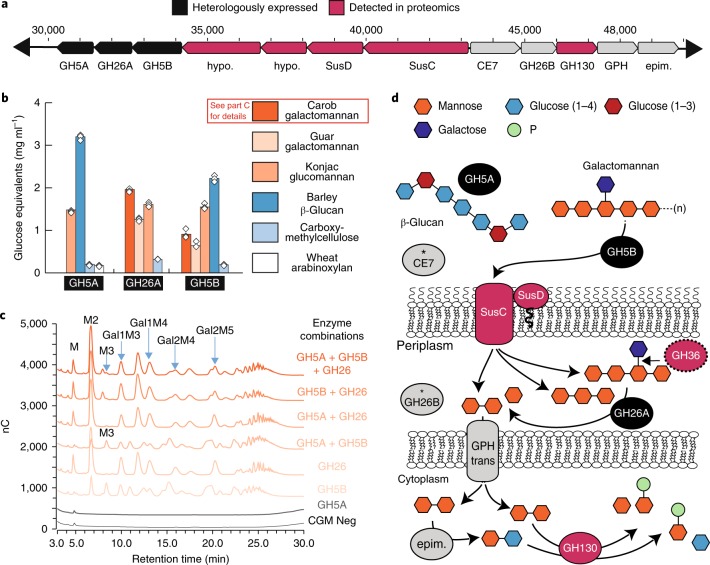
Biochemical confirmation of PUL predictions. **a**, Gene organization of a predicted mannan PUL identified in *Prevotella* sp. PREV32, which was selected for in-depth biochemical characterization. Gene abbreviations: epim., mannobiose-2-epimerase; GPH, glycoside-pentoside-hexuronide transporter; hypo., hypothetical. **b**, Purified proteins incubated with six plant polymers highlighted the differential use of plant polymers by glycoside hydrolases. The bars represent an average of three replicates (white diamonds), are coloured by plant polymer and represent reducing ends (glucose equivalents) recovered. **c**, Incubation of purified enzymes with carob galactomannan (CGM) identified peaks matching elution times of standards, including mannose (M), β-1,4-manno-oligosaccharides (M2–M3) and manno-oligosaccharides substituted with (X) galactose residues (Gal(X)M(X)). The spectra represent one run of three replicates. ‘CGM neg’ is a negative control with CGM and no enzymes. nC, nanocoulombs. **d**, A hypothetical model, based on predicted protein locations^70,71^ and biochemical data, depicting a process in which galactomannan is bound via outer membrane lipoproteins (SusCD), hydrolysed via GH5B to shorter manno-oligomers (with and without galactosylations) and imported into the periplasm. Galactose residues could be removed by a GH36 (detected in proteomics and located in another part of the genome), providing manno-oligomers as substrate for the GH26 enzyme. Hydrolysed galactomannan products (mannobiose and mannose) could be further transported into the cytoplasm by the GPH transporter (trans). In the cytoplasm, the GH130 enzyme can process either mannobiose or mannosyl-glucose generated from the epimerase in the PUL. Based on the substrates identified for GH5A, it could have a role in paving the way for GH5B access to mannans in complex substrates. Other enzymes in this PUL (indicated by the asterisks) include CE7, which could contribute to the degradation of acetylated mannans, and GH26B, which could be complementing GH26A.

To further understand the role of PULs in the rumen ecosystem, we selected one highly expressed PUL for heterologous expression and biochemical analyses (Fig. 4). This PUL from the PREV32 genome (most closely related to *P.* *ruminocola*) has gene organization similar to a *Bacteroides cellulosilyticus* WH2 PUL, with two GH5 enzymes and one GH26 co-localized with the SusCD-like lipoproteins^29^ (Fig. 4a, Supplementary Dataset 1 and Supplementary Table 5). Co-localized, but in the reverse coding sense, we observed a CE7, GH26, GH130, mannobiose-2-epimerase and a glycoside-pentoside-hexuronide sugar transporter, which are probably part of the same PUL (Fig. 4a). Metaproteomics detected peptides from the SusCD-like proteins, two hypothetical proteins and the GH130 (Fig. 4a). Using proteomic and genomic data, we conservatively predicted that this PUL could degrade mannan and import its products, where the GH130 enzymes in the cytoplasm would release mannose and glucose for incorporation into glycolysis.

To test this prediction, we synthesized and expressed the two GH5 (GH5A and GH5B) and the GH26 enzymes encoded downstream from the proteome-detected lipoproteins (Fig. 4a) and measured their activity on various mannan and glucan substrates (Fig. 4b). As expected from proteomic predictions, all enzymes were individually active on mannan substrates, with GH26 displaying the highest activity on galactomannan and glucomannan. GH5B also displayed typical endomannanase activity, releasing mannotriose, mannobiose and galactosylated manno-oligomers from galactomannan. By contrast, GH5A was the most active on β-glucan and also had weak activity on carboxymethylcellulose and arabinoxylan, consistent with the classification of this GH5 into subfamily four^30,31^. This approach complemented our proteomics, but also provided more resolved functional predictions, including the specific compounds and enzyme cleavage mechanisms. For instance, although we predicted that the GH5 enzymes could be active on mannan, biochemical results revealed different substrate specificities for GH5A and GH5B (Fig. 4b). The GH5A enzyme was not active on all mannan-backbone-containing substrates, but was more active on β-glucan/xylan-containing hemicellulose substrates, suggesting a potential role in removing these polymers from plant fibres to liberate mannan for utilization.

The biochemical approach also demonstrated that, in combination, these proteins (GH5A, GH5B and GH26) have a different mode of action than the proteins functioning alone. GH26 alone was responsible for most of the mannobiose release from galactomannan; however, together with GH5B, the two enzymes produced a synergistic effect in which the GH26 enzyme could also produce differential metabolites produced from products released by GH5B (that is, mannotriose and other unidentified products; Fig. 4c). The additional resolution into substrate specificity and enzymatic interactions revealed the versatility of this multiple-substrate-targeting PUL and the multiple enzymes required for breaking down complex mannan substrates. Using a combination of proteomics and specific enzyme activity measurements, we developed a working model for how this PUL can break down mannan and generate sugars for central metabolism (Fig. 4d). Future isolate investigations will help to refine this model and continue to shine light on the contribution of PULs in the rumen.

**Fig. 5:**
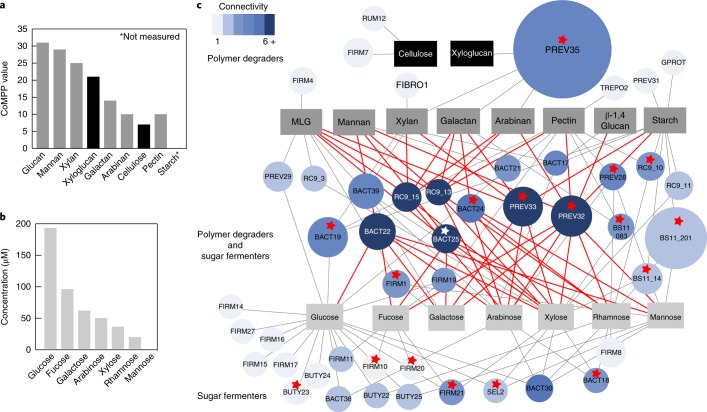
Network analysis of plant carbon degradation. **a**, CoMPP value of detected carbon polymers in winter rumen fluid, representing the relative abundance of polymers in the winter diet sample. **b**, Concentration of 5C and 6C sugars measured by ^1^H NMR. **c**, Network nodes represent carbon substrates (rectangles) and MAGs (circles; 47 total). The abbreviations and antibodies used for carbon substrates are provided in Supplementary Dataset 1, Supplementary Table 7. MAGs are sized by total coverage and coloured by connectivity. Nodes are connected if proteins for degrading the substrate were detected in metaproteomics and were unique to the genome. Polymers are at the top in dark grey and sugars are at the bottom in light grey. The red stars indicate that proteins for SCFA production were detected from that MAG in metaproteomics. Edges from highly connected (more than six connections) genome nodes are outlined in red. MAGs are labelled by taxonomic assignment, with established genera named by genus and previously undescribed genomes labelled by phylum. BACT; Bacteroidetes; BUTY, *Butyrivibrio* sp.; FIBRO, *Fibrobacter* sp.; FIRM, Firmicutes; GPROT, Gammaproteobacteria; PREV, *Prevotella* sp.; RUM, *Ruminococcus* sp.; SEL, *Selenomonas* sp.; TREPO, *Treponema* sp. Names and abbreviations are also provided in Supplementary Dataset 1, Supplementary Table 1.

### Rumen metabolites are coordinated to PUL substrate predictions

Mannan was one of the most highly abundant substrates detected based on carbohydrate microarray polymer profiling (CoMPP), which compares the relative abundances of plant polymers (Fig. 5a). However, we only detected trace amounts of mannose, the monomeric sugar unit making up this polymer in our nuclear magnetic resonance (NMR) metabolite data (Fig. 5b). Other highly abundant polymers also have a high abundance of the corresponding sugars (for example, xyloglucan and xylose, mixed-linked glucan/cellulose and glucose) (Fig. 5). This suggests that the mannan degraders that are active in this ecosystem could use a ‘selfish mechanism’ by importing and degrading larger manno-oligos within the cell, instead of releasing mannose to the environment. Our working model based on a combination of proteomics, metabolite data and biochemical investigations supports this selfish mechanism hypothesis (Fig. 4d) and may contribute to the success of the Bacteroidetes in the rumen and in the human gut^32^.

To better examine the microorganisms responsible for carbohydrate degradation in the rumen, we linked all of the PULs detected in proteomics to metabolite data collected from NMR and CoMPP. Many PULs detected in metaproteomics were predicted to degrade hemicellulose polymers (for example, mixed-linked glucans and xylan) (Fig. 3c). Correspondingly, hemicellulose polymers (for example, xylan, mannan and xyloglucan) were the most abundant plant polymers detected (Fig. 5a, Supplementary Fig. 4). With the exception of mannan, we also detected a high concentration of monomeric sugar substituents making up these polymers (for example, xylose and glucose) (Fig. 5b), suggesting that other hemicellulose PULs may be operating to release these sugar constituents to the rumen fluid.

### Microbial trophic network of uncultivated taxa supports rumen carbon degradation

In anaerobic ecosystems, carbohydrate decomposition is performed through interconnected microbial metabolisms^33^. To expose metabolic networks in the rumen, we reconstructed the carbon degradation network based on coordinated expression data and carbon metabolite pools. Based on linkages to specific substrate classes, genomes were assigned to one of three trophic levels in the carbon food chain: (1) recalcitrant plant polymer degradation; (2) mixed polymer degradation and sugar fermentation; or (3) exclusive sugar fermentation (Fig. 5c).

Genomes from which we solely detected proteins for the degradation of more recalcitrant polymers (for example, cellulose and xyloglucan, black substrates) included *Prevotella* species (PREV35 and PREV31) and members of the Ruminococcaceae family, including a genus defined here, *Candidatus* (*Ca*.) Vansoestibacter (FIRM7), and a *Ruminococcus* sp. genome (RUM12) (Fig. 5c). These findings are consistent with rumen *Prevotella* and *Ruminococcus* isolates that degrade these polymers in the laboratory. PREV35 was the most abundant genome and was the only genome from which we detected PUL genes (GH31) for xyloglucan degradation in proteomics. This suggests that the unique usage of this substrate may confer dominance of PREV35 in the rumen. Cellulose-degrading enzymes (GH48) were only detected from two MAGs (FIRM7 and RUM12) and were probably encoded within cellulosomes (Fig. 5c and Supplementary Discussion). The rarity of cellulose and xyloglucan degraders suggests that these lineages play an essential role in the conversion of complex plant material into more readily degradable substrates, fuelling the metabolisms of other sugar and polymer degraders.

Unlike exclusive polymer degraders, the next trophic level is composed of metabolically more versatile members. Eighteen of the 20 genomes in this trophic level are members of the Bacteroidales and encode PULs to degrade plant polymers (Fig. 3). Six Bacteroidetes genomes (dark blue in Fig. 5c) are co-expressing the genes for the utilization of six or more substrates (polymers and sugars) and are consequently referred to as metabolic hubs (Fig. 5c). Notably, these hub genomes mostly belong to taxa that were enriched in our winter rumen fluid (Supplementary Fig. 1), previously seen to be prevalent across ruminant animals^2^, and either recently genomically sampled (RC9 gut group)^5,7^ or are described here (*Ca*. Aleyska and *Ca*. Hungataceae). We note that one of these hub genomes, PREV32, was also selected for PUL biochemical characterization (Fig. 4). Proteins expressed from the six metabolic hub genomes can degrade 13 of the 15 detected polymer or sugar carbon substrates (not cellulose or xyloglucan). Within this trophic level, closely related organisms seem to partition resources to co-exist. For instance, mannan degradation is the most widely encoded polymer degradation trait (Fig. 2), but in metaproteomics, we detected peptides from mannan-degrading enzymes from only five genomes (Fig. 5c). These results show that, although rumen microbiota are largely functionally redundant with regards to metabolic potential (Fig. 2), metaproteomics demonstrated specialization into functional guilds defined by substrate.

The third trophic level is composed of obligate fermenters of five-carbon and six-carbon sugars, organisms that are not expressing glycoside hydrolases to degrade plant polymers but, instead, express the isomerase or kinase for the incorporation of specific sugars into the central metabolism. Glucose is the most abundant sugar and also the most highly connected metabolite node, with 12 genomes expressing glucose-6-phosphate isomerase for its utilization. However, only one hub genome is expressing genes for glucose degradation, implying that these versatile organisms tend to rely on uncommonly used substrates. Unlike in the first two trophic groups, more than 80% of this group are Firmicutes. Seven of these Firmicutes genomes belong to three genera: *Buytrivibrio*, *Ruminoclostridium* and *Selenomonas*. Surprisingly, all *Butyrivibrio* genomes encode glycoside hydrolase genes for hemicellulose degradation that were not detected in our metaproteomes. This may reflect the ability of the Bacteroidetes PULs to outcompete *Butyrivibrio* carbon degradation mechanisms in the moose rumen on a high-lignocellulose diet. The remaining 11 genomes are metabolically characterized representatives of previously unknown Bacteroidetes and Firmicutes. These results demonstrate the clear contribution of known and previously undescribed lineages to active sugar fermentation in the rumen (Fig. 5 and Supplementary Discussion).

Microbial carbon degradation in the largely anoxic rumen results in the production of butyrate, acetate and propionate, which are critical SCFAs that can contribute up to 80% of the host’s energy^1^. Although SCFA production was encoded in nearly all genomes (Fig. 2), our proteomics data highlighted contributions by 17 members (Fig. 5, red star), including known SCFA producers (for example, *Butyrivibrio* and *Prevotella*) and previously unknown players in this food web (for example, *Ca*. Aleyska, RC9 gut group and BS11). Many of the SCFA-producing bacteria are conserved across ruminant animals consuming various different plant diets (for example, RC9 gut group, uncultivated Prevotellaceae, uncultivated Ruminococcaceae and BS11)^2,14^. Our genome-resolved proteomic inferences provide a glimpse into the metabolic flexibility of these taxa, which may contribute to their maintenance across ruminants and dietary regimes.

### Viral infections are a key modulating factor of rumen microbial ecosystems

We identified 1,907 viral contigs (>10 kb), 93 of which were circular (closed) viral genomes. We clustered these contigs into 810 viral populations and taxonomically classified them using NCBI RefSeq v75 (ref. ^34^) and viral genomes from a previous rumen metagenomics data set^10^. This analysis enabled us to detect 148 viral genera, 75% of which lack a database reference sequence and represent previously unknown viral genera (Supplementary Fig. 5A). Some of these genera in moose (35) also contain viruses from the cow rumen, thereby representing conserved rumen viruses. This shows that different animals share viral genotypes.

To examine the role of these viruses in the rumen, we mined our metaproteomics data for expression of viral genes, detecting a total of 64 viral proteins from 53 different viral contigs (Supplementary Fig. 5B). Most viral proteins (80%) had no known functions and were identified as hypothetical proteins, whereas the remaining were largely structural proteins, such as capsids (Supplementary Dataset 1, Supplementary Table 6). This signifies that these previously enigmatic components are active players in moose rumen microbial communities.

**Fig. 6:**
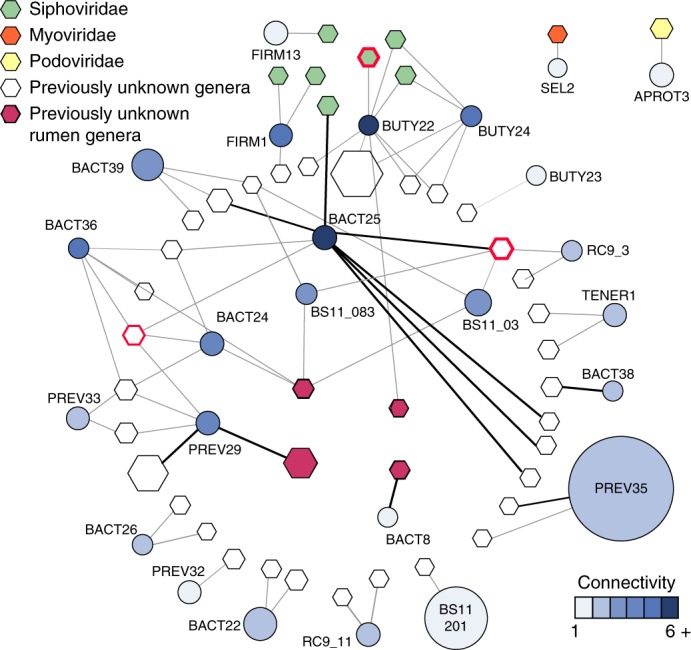
Host–viral interaction network. Viral genomes (hexagons) are connected to predicted microbial host genomes (circles) by edges. All genomes (viral and microbial) are sized by abundance across data sets. Viral genomes or contigs are coloured by taxonomic assignment. Microbial host MAGs are coloured by connectivity noted on the bottom right. An edge was drawn if a confident link could be established by tetranucleotide frequency (grey edges) or protospacers within the CRISPR–CRISPR-associated protein (Cas) systems (black edges). The length of the edge has no meaning. Viral genomes or contigs with proteins detected in proteomics are outlined in red.

To further understand the role of these viruses in the rumen, we examined our viral contigs for auxiliary metabolic genes (AMGs) to determine genes that are present in microbial metabolic pathways and not typically associated with viral function^35^. We detected very few AMGs, with no genes detected to redirect carbon metabolism, contrary to previous findings in rumen viruses^12^ (Supplementary Discussion). Based on our findings, it is more likely that when moose are consuming a winter diet, rumen viruses are predominantly affecting carbon cycling in a top-down manner, by infecting ecologically critical microorganisms. To evaluate virus–host interaction dynamics, we used similarities in virus–host genome tetranucleotide frequency and clustered regularly interspaced short palindromic repeats (CRISPR) protospacer matches to viral genomes. Host genomes were predicted for 113 viral contigs spanning 4 of the 9 phyla sampled. Forty-six of these viral contigs could be directly linked to a specific MAG, including the most dominant and active carbon-degrading populations: *Prevotella* sp. (PREV35) and BS11 MAGs (Fig. 6). These findings show that viral predation could indirectly affect all trophic levels of carbon processing, targeting organisms that use complex polymers, such as xyloglucan (PREV35), hemicellulose polymers (BACT25) and hemicellulosic sugars such as xylose (BS11)^14^.

## Conclusion

Here, we provide a phylogenetic framework and naming system for hundreds of genomes from at least 12 previously undefined lineages in Bacteroidetes, Firmicutes and Tenericutes that lack a cultivated representative to the genus or family level. Many of these lineages are not unique to the rumen, but are present across other host ecosystems. In particular, Bacteroidetes PULs are known to be active in the human gut^21–23^, soils^24^ and the deep ocean^36^. Collectively, our integrated metabolite proteomic results parse microorganisms into substrate niches and reveal the metabolic triaging of plant biomass among different microbial genotypes. Furthermore, our rumen virome and coupled proteome data identify viruses that are conserved across animal gastrointestinal tracts and show that these viruses actively infect dominant carbohydrate-degrading microorganisms to modulate gastrointestinal carbon cycling. In conclusion, the themes identified here extend beyond the rumen to basic ecology of other anoxic carbon-rich ecosystems. Our data provide a foundation to model and develop a predictive understanding of metabolic exchanges and trophic structure relevant to carbon degradation in anoxic ecosystems.

## Methods

### Experimental design, sequencing, assembly and genome reconstruction

The moose used in this initial study are the only two rumen-fistulated moose in the world. This surgical procedure provided unparalleled access to rumen fluid samples from live moose as they were consuming and digesting food. These moose (female, *Alces alces gigas*), both 12 years of age, were monitored over the course of 1 year, as they consumed three seasonal diets (spring, summer and fall/winter) in Alaska and a control pellet diet (Institutional Animal Care and Use Committee protocol no. 754207-2). After a 1-week diet adjustment period, each moose was sampled three times on the diet over 1 week. Microbial community analyses using 16S rRNA gene sequencing determined that sample replicates per diet treatment were statistically similar and independent of host. These findings were published previously in *ISME Journal*^14^. From these previous analyses, we demonstrated that uncultivated Bacteroidetes were enriched in the winter rumen fluids. To uncover the physiological roles and substrate preferences for these uncultivated Bacteroidetes prevalent in the winter rumen fluid, here, we deeply sequenced four metagenomes (total 53.8 Gbp) and conducted three metaproteomes (over 19,000 peptides) from one moose winter rumen fluid sample (Supplementary Fig. 1). This approach allowed us to sequence a single sample deeply to recover genomes from low abundant, but perhaps functionally important, members. The sequencing depth allocated to a single sample is much larger than most rumen sequencing projects to date (for example, Brulc et al.^8^, Wallace et al.^37^, Lopes et al.^38^ and Svartstrom et al.^7^). This approach was critical, as our goal was to create a genome-resolved database to which we could map the metaproteome data, enabling the discovery of a carbon degradation food web (incorporating genome, enzyme and metabolite insights) prevalent in the rumen.

Previously, we have reported details of the microbial community assembly and sampling^14^ and only summarize the methods here (Supplementary Fig. 1). Rumen fluid samples were first centrifuged at low speed (6,000 *g*) to separate plant material (the pellet). The supernatant was then sequentially filtered onto a 0.8-μm filter (F08) and a 0.2-μm filter (F02). Half of the pellet and half of the filters were submitted for sequencing. The remaining halves were frozen and sent to the Environmental and Molecular Sciences Laboratory (EMSL) for metaproteomics. In addition, the post-0.2-μm filtrate was concentrated and submitted for sequencing of small cells and viruses. Illumina sequences from DNA extracted from each of the four samples were assembled individually, then co-assembled^39^. Assembly statistics are reported in Supplementary Table 2. Genome fragments were binned using multiple approaches. Individual assemblies and co-assembled metagenomes were binned individually using emergent self-organizing map analysis, MetaBAT, and a combination of phylogenetic signature and guanine-cytosine content (manual binning)^40–42^. For the emergent self-organizing map, the primary map structure was established using 2-kb fragments (all fragments >10 kb were subdivided into 2-kb fragments). For MetaBAT binning, we used the very sensitive setting for all scaffolds >2 kb. Manual binning was performed as previously described^14,40^. The code and further software descriptions for metagenome assembly are available online https://github.com/TheWrightonLab/metagenome_assembly.

### Genome dereplication, annotation and proteomics

The same genome was often sampled multiple times (different size fractions or binning methods); thus, we selected the highest scoring genome as the unique genotype^43^. We first dereplicated our genomes by generating an alignment of scaffolds within one genome individually against scaffolds of all other bins at 98% nucleotide identity or greater. Genomes were then grouped at >50% similarity level and the best representative was chosen based on a scoring system of single copy genes. The score is equivalent to the number of archaeal or bacterial single copy genes (−2) times the number of multiple single copy genes^39^. In the case of a tie, the genome with the greatest nucleotide information (length) was chosen. For each unique genome, we report the recovery of transfer RNA, rRNA, the number of scaffolds, guanine-cytosine content and assembly quality statistics to determine quality as proposed by the Genomic Standards Consortium^15^ (Supplementary Table 2).

Details of the annotation and proteomic analysis were published previously^14^ and are briefly summarized here. Genes were predicted using MetaProdigal^44^ and annotated using USEARCH40 (ref. ^45^) to Kyoto Encyclopedia of Genes and Genomes (KEGG)^46^, UniRef90 (ref. ^47^) and InterproScan^48^, with single and reverse best-hit matches greater than 60 bits reported. These predicted proteins formed a database that was searched via MSGF+ with collected two-dimensional liquid chromatography–tandem mass spectrometry data from the extracted biomass on the filters and plant pellet as described previously^49^. Briefly, filters were extracted using SDS-lysis buffer, 100 mM Tris/HCl and sonication at 40% amplitude for 20 s and repeated. This is consistent with best practices for proteomic extraction methods in the gut^26^. Data were collated using an in-house program, imported into a SQL server database, filtered to ~1% false discovery rate (peptide to spectrum level) and combined at the protein level to provide unique peptide count (per protein) and observation count (that is, spectral count) data. Protein identification was based on two or more unique peptides per protein. All scripts used for metagenome annotation are available online https://github.com/TheWrightonLab/metagenome_annotation.

### Phylogenetic analyses

Phylogenetic analysis was performed using two different single copy marker genes, the 16S rRNA gene and a syntenic block of 16 universal ribosomal proteins (L2–L6, L14–L16, L18, L22, L24, S3, S8, S10, S17 and S19)^43,50^. All methods were used for validation of phylogeny when possible; however, 16S rRNA gene sequences were only recovered in 16 MAGs. Genomes from other rumen metagenomic data sets (345 genomes from the UBA, Hungate 1000 and others)^5–7,9,16^ were recruited for the phylogenetic resolution of monophyletic lineages containing only genomes that have not been taxonomically assigned. Ribosomal proteins were found in reference genomes using metaprodigal and the annotation pipeline described above. Individual ribosomal proteins were aligned separately by phylum using MUSCLE^51^, with default parameters and then manually curated to remove end gaps. Individual protein alignments were concatenated in Geneious version 7 (ref. ^52^). Phylogenetic analyses for ribosomal proteins and 16S rRNA genes were inferred using RAxML^53^, under the PROTGAMMALG method for protein sequences and GTRGAMMA method for 16S rRNA genes^50^. All trees were rooted to genomes in the Actinobacteria phylum, with the exception of that in Fig. 1, which was rooted to the Euryarchaeota phylum. Complete concatenated protein trees by phylum are available in Nexus format as Supplementary Dataset 2–10. The complete 16S rRNA gene trees for all MAGs with 16S rRNA gene sequences are provided in Newick format in Supplementary Datasets 14–26. All trees were visualized using iTOL^54^.

### Requirements for proposing nomenclature

For assigning names to our MAGs, we required at least three genomes, which are monophyletic by concatenated ribosomal protein tree analyses with bootstrap support of >70. If present, 16S rRNA gene phylogeny needed to be congruent with concatenated ribosomal protein analyses. The use of *Ca.* denotes names proposed for lineages resolved here^55^. A complete naming description and justification for all taxonomic groups recovered is provided in the Supplementary Discussion.

### Detection and substrate prediction for PULs

PULs are composed of SusCD-like proteins surrounded by glycoside hydrolases, transporters and carbon recognition proteins used for the import and degradation of complex carbohydrates. To comprehensively profile the PULs from all of our Bacteroidetes genomes, we first performed a general search for co-localized SusCD-like proteins. As SusC is a TonB-dependent receptor and commonly found in most organisms, we scanned our genome annotation files for Pfam identifiers associated with SusD-like proteins (PF07980, PF12741, PF12771 and PF14322)^27,56,57^. If genes with the Pfam identifiers were co-localized with a TonB receptor or annotated SusC-like protein, we looked at the surrounding genes for a CAZyme (identified via dbCAN^58^) or peptidase within six open reading frames in the same gene orientation. This resulted in a total of 198 putative PULs. These PULs were all compared to experimentally verified PULs in PULDB and other recently characterized PULs in the literature^21,22,36^. A majority (69%) of our PULs had similar CAZy families and gene organization to biochemically defined PULs, allowing us to predict substrates. PULs with no previous biochemical characterization were annotated here as substrate unknown (Supplementary Dataset 1, Supplementary Table 5). Many other recent studies have reported PULs that do not contain any CAZyme nearby, but just contain a SusCD-like pair^6,16^. Because we were interested in linking genomes to substrates, we used more conservative estimates of PUL substrate calls. For every PUL that we recovered containing a CAZyme or peptidase within six open reading frames, we report: (1) the taxonomic assignment of the genome that it was assigned to (and the location of this PUL or genome in a publicly available database); (2) the gene order of the glycoside hydrolase families in the PUL; (3) substrates targeted by similar biochemically characterized PULs (and associated references); and (4) the number and identity of PUL-associated proteins detected in our metaproteomics. This information can be found in Supplementary Table 5.

To predict the substrate for each of these PULs, we used multiple approaches. Many PULs had a unique organization of glycoside hydrolases or peptidase genes surrounding the SusCD-like proteins and could not be confidently assigned to a substrate via putative functional gene annotations. All glucan PULs containing GH16, GH30 and a β-glucosidase (that is, GH3) were categorized as a putative mixed-linked glucan PUL^36^. We did not characterize any PUL as cellulolytic, as this has never been experimentally verified by cultured strains^59^. However, for all PULs that contained an endoglucanase (GH5 and GH9), β-glucosidase (GH3) and/or cellobiose phosphorylase (GH94), in the absence of glycoside hydrolases that target the side chains of branched glucans, we specifically identified them as β-(1,4)-glucan PUL. A detailed list of glycoside hydrolases included in our PUL substrate calls can be found in Supplementary Dataset 1, Supplementary Table 5.

### Heterologous expression, purification and characterization of PREV32 genes

The amino acid sequences encoded by the predicted genes of the putative mannan-targeting PUL of PREV32 (PREV32_scaffold_288_28-34) were codon-optimized for *Escherichia coli*, synthesized and cloned into pET-28a(+) plasmids by GenScript. PREV32_scaffold_288_28 and PREV32_scaffold_288_30, GH5A and GH5B, respectively, were synthesized without predicted signal peptides^60^. Plasmids were transformed into *E.* *coli* One Shot BL21 Star cells (Thermo Fischer Scientific) and an overnight pre-culture was inoculated to 1% in 200 ml lysogeny broth with 50 mM kanamycin, incubated at 37 °C, with shaking at 180 revolutions per minute. Expression was induced at an *A*_600 nm_ of 0.6 by addition of isopropyl-β-d-thiogalactopyranoside to a final concentration of 0.4 mM. The culture was incubated at 37 °C, 180 r.p.m. for 4 h, and cells were harvested by centrifugation (4,500 *g* for 20 min). Cells were washed once in 50 mM Tris-HCl and 0.2 M NaCl, pH 8.0 (room temperature), before resuspension in 5 ml 50 mM Tris-HCl, 0.2 M NaCl and 10 mM imidazole, pH 8.0, with 1× BugBuster (Merck Millipore). Cell lysate was obtained by centrifugation (4,500 *g* for 20 min) and purified using 5 ml HisTrap columns (GE Healthcare), using a linear gradient of imidazole to 0.5 M. The buffer was changed to 50 mM Tris-HCl and 0.2 M NaCl, pH 8.0, by repeated concentration and dilution, before protein concentrations were calculated from *A*_280 nm_ using the estimated extinction coefficient of the expressed protein sequences.

Enzymatic assays were performed in a 96-well plate and contained 20 mM BisTris buffer pH 6.0 (at 40 °C) and 0.5 mg ml^–1^ glucomannan (konjac, low viscosity, Megazyme), galactomannan (carob, Megazyme), galactomannan (guar, Megazyme), β-glucan (barley, medium viscosity, Megazyme), carboxymethylcellulose (low viscosity, Sigma), arabinoxylan (wheat, Megazyme) or 0.1 mg ml^–1^ carboxymethyl-curdlan (Megazyme). Reactions were pre-heated (40 °C for 10 min) in a Thermomixer C incubator with a heated lid (Eppendorf), before addition of enzyme to 0.5 µM (final reaction volume: 100 µl) for further incubation (60 min). The reactions were stopped by addition of 1% DNS reagent (100 µl)^61^, and the sealed plate was heated to develop colour (95°C for 20 min). Heat-treated samples (150 µl) were transferred to a fresh plate after chilling on ice and *A*_540 nm_ was measured in a Multiscan FC Microplate Photometer (Thermo Scientific). Released reducing ends were quantified against a standard curve of glucose. Degradation of insoluble ivory nut mannan (5 mg ml^–1^, Megazyme) was examined using an extended incubation time of 3 h. The reactions were centrifuged (14,000 *g* for 5 min) and the supernatant was further examined by DNS assay, as described above.

To analyse products from carob galactomannan, reactions were stopped by addition of NaOH to a final concentration of 0.1 M. Products were analysed by high-pH anion-exchange chromatography with pulsed amperometric detection on a Dionex ICS-5000 system with a CarboPac PA1 column, at a flow rate of 0.25 ml min^–1^. Oligosaccharides were eluted in a multistep linear gradient going from 0.1 M NaOH to 0.1 M NaOH – 1 M sodium acetate. The products were identified using standards of gluco-oligosaccharides and manno-oligosaccharides (Megazyme), and degradation products were identified by matrix-assisted laser desorption/ionization–time of flight from guar galactomannan by an in-house-produced bacterial GH26.

### Metabolic reconstruction and network analyses

To construct the metabolic heat map, we scanned our genome annotations for genes involved in specific metabolisms. For polymer degradation, we performed a Pfam scan, as previously described^14^, and looked for glycoside hydrolases involved in the degradation of specific polymer substrates detected with our CoMPP analyses, following a similar structure to the PUL analyses. For the ability to degrade cellulose, a full cellulosome needed to be detected. For sugar utilization, we required the full pathway of all rare 6C sugar monomers (fucose, mannose and rhamnose) and at least 6 of 9 Embden–Meyerhof–Parnas (EMP) pathway genes. For 5C sugars, we required the presence of the specific isomerase and epimerase and the full pentose phosphate pathway. For fermentation end products, we looked for all possible pathways and required two-thirds of the genes to be present for the organism to be capable of producing it. All genes and associated Enzyme Commission numbers used in these analyses can be found in Supplementary Dataset 1, Supplementary Table 4.

Beyond genomic potential, we wanted to evaluate which of these metabolic traits were active. For polymer degradation, at least one gene with more than one unique peptide in the polymer degradation mechanism was required (for example, for cellulose, one of the cellulosome modules needed to be detected in metaproteomics; for PULs, one of the co-localized genes needed to be turned on). Because all sugars are eventually fermented via the EMP pathway, we looked for the expression of specific sugar isomerases or kinases (rhamnose isomerase, galactokinase, mannose-6-phosphate isomerase, xylose isomerase, arabinose isomerase, glucose-6-phosphate isomerase and fucose isomerase) to determine the substrate. We also required the detection of one downstream EMP pathway gene. Glucose-6-phosphate isomerase was utilized for determining whether an organism was expressing glucose metabolism genes. However, because galactose enters the EMP pathway at this step, we also required peptides for glucokinase or a glucose-specific transporter to determine whether it was also using glucose. If we detected proteins for the degradation of a plant compound or a sugar isomerase, we created a connection between genome nodes and substrate nodes^62^. The network was visualized in Cytoscape 3.4.0 (ref. ^63^). The number of edges connected to a node determined connectivity.

### Carbohydrate microarray profiling

Whole-rumen contents from winter rumen fluid samples (*n* = 6) were vortexed and pooled to equal weights (10 mg). Cell-wall glycans were sequentially extracted, using 50 mM diamino-cyclo-hexane-tetra-acetic acid, pH 7.5, and 4 M NaOH with 1% v/v NaBH_4_, which are known to predominantly release pectins and non-cellulosic polysaccharides, respectively. For each extraction, 300 μl solvent was added to 10 mg rumen samples and then incubated at room temperature with shaking for 2 h. Samples were then centrifuged at 2,700 *g* for 10 min to remove cell debris. Retained supernatants were diluted sequentially (1/2,1/5,1/5,1/5) in microarray printing buffer (55.2% glycerol, 44% water and 0.8% Triton X-100), and the four dilutions were printed in quadruplet onto nitrocellulose membranes using a non-contact microarray robot (Arrayjet, Roslin). Thus, every replicate was represented by a 16-spot subarray (four concentrations and four printing replicates). Arrays were probed with monoclonal antibodies or carbohydrate-binding modules^64^, scanned and uploaded into Array-Pro Analyzer 6.3 analysis software. The maximal mean spot signal was set to 100% and all other values within that data set were adjusted accordingly. A mean spot signal minimum was set as 5%. Results from six rumen fluid samples were averaged to calculate a mean abundance of individual plant polymers.

### Discovery of viral contigs from metagenomes

VirSorter was used to recover viral contigs based on the identification of viral hallmark genes, strings of hypothetical proteins and other viral signatures, as previously described^65^. Each of the four metagenomic data sets were used in a separate VirSorter run. We only considered VirSorter categories 1 and 2 (and 4 and 5, the provirus equivalents of categories 1 and 2), which are the categories with the highest confidence^65^. The data set of detected viral populations was manually curated to a final set of 1,907 viral contigs by ensuring consistency with a viral origin in the Pfam annotation, as described previously^66^. This viral database was dereplicated by clustering at 95% nucleic acid identity with Cd-Hit v4.6 (ref. ^67^) to generate the final 810 unique viruses for classification.

### Taxonomic classification of viral contigs via vContact

A network-based gene content classification was used to place the 810 viruses in the context of known viruses^65,66^. Briefly, predicted proteins from viral contigs were clustered with predicted proteins from viruses in the NCBI RefSeq database (v75, June 2016)^34^ based on an all-versus-all BLASTp search with an E-value threshold of 10^−4^, and protein clusters were defined with the Markov clustering algorithm, as previously described^65,66^. vContact was then used to calculate a similarity score for each contig–genome or genome–genome pair (https://bitbucket.org/MAVERICLab/vcontact, accessed September 2016)^68^. The stringency of the similarity score was evaluated through 1,000 randomizations by permuting protein clusters or singletons (proteins without significant shared similarity to other protein sequences) within pairs of genomes and/or contigs having a significance score of ≤1 (a negative control)^69^. Subsequently, pairs of genomes and/or contigs with a similarity score of >1 were clustered into viral clusters with the Markov clustering algorithm with an inflation of 2, as previously described^66^. The resulting network was visualized with Cytoscape software (version 3.4.0; http://cytoscape.org/), using an edge-weighted spring-embedded model, which places the genomes and/or contigs that share more protein clusters in closer proximity in the display. Reference sequences from the RefSeq genomes that co-clustered with the 810 rumen viruses in this study were used to predict viral taxonomy. A last common ancestry approach was applied to all reference sequences containing viral clusters in which RefSeq genomes were clustered. Taxonomic affiliation of rumen viruses was based on the taxonomy of the RefSeq genomes. If the RefSeq genomes differed in taxonomy, the highest taxonomic level in common for the reference sequences was retained. If viral clusters exclusively contained rumen viruses from this study, the viral cluster was considered a candidate genus.

Predicted viral proteins from unique genomes were searched for as described for MAGs^48^. Viral protein identification was first compared to the microbial metagenomes and overlapping hits were subtracted. To be more sensitive and to enable the detection of more ‘rare’ viruses, we considered hits for viral proteins based on one or more unique peptides per protein.

### Detection of auxiliary metabolic genes on viral contigs

To determine whether viral contigs had AMGs, we annotated non-prophage viral contigs identified in VirSorter as previously described^40^. Briefly, we used MetaProdigal^44^ to call genes and annotated proteins using USEARCH40 (ref. ^45^) to KEGG^46^, UniRef90 (ref. ^47^) and InterproScan^48^, with single and reverse best-hit matches greater than 60 bits reported. We manually searched these annotations for glycoside hydrolases and the 75 known AMGs present in the KEGG database^12^.

### Reporting Summary

Further information on research design is available in the Nature Research Reporting Summary linked to this article.

### Code availability

A description of all software, including scripts and commands, used for the analyses in this paper can be found at https://github.com/TheWrightonLab/.

## Supplementary information

Supplementary Information Supplementary Discussion, Supplementary Figures 1–5, Supplementary References, Supplementary Dataset legends.

Reporting Summary

Supplementary Dataset 1 Supplementary Tables 1–7.

Supplementary Dataset 2 Concatenated ribosomal protein tree of 16 ribosomal proteins for all MAGs in the Tenericutes phylum, with reference sequences in Newick format.

Supplementary Dataset 3 Concatenated ribosomal protein tree of 16 ribosomal proteins for all MAGs in the Firmicutes phylum, with reference sequences in Newick format.

Supplementary Dataset 4 Concatenated ribosomal protein tree of 16 ribosomal proteins for all MAGs in the Spirochaetes phylum, with reference sequences in Newick format.

Supplementary Dataset 5 Concatenated ribosomal protein tree of 16 ribosomal proteins for all MAGs in the Fibrobacteres phylum, with reference sequences in Newick format.

Supplementary Dataset 6 Concatenated ribosomal protein tree of 16 ribosomal proteins for all MAGs in the Saccharibacteria (TM7) phylum, with reference sequences in Newick format.

Supplementary Dataset 7 Concatenated ribosomal protein tree of 16 ribosomal proteins for all MAGs in the Euryarchaeota phylum, with reference sequences in Newick format.

Supplementary Dataset 8 Concatenated ribosomal protein tree of 16 ribosomal proteins for all MAGs in the Lentisphaera phylum, with reference sequences in Newick format.

Supplementary Dataset 9 Concatenated ribosomal protein tree of 16 ribosomal proteins for all MAGs in the Proteobacteria phylum, with reference sequences in Newick format.

Supplementary Dataset 10 Concatenated ribosomal protein tree of 16 ribosomal proteins for all MAGs in the Bacteroidetes phylum, with reference sequences in Newick format.

Supplementary Dataset 11 Full ribosomal protein S3 tree in Newick format shown in Figure 1.

Supplementary Dataset 12 Full concatenated ribosomal protein tree of 16 ribosomal proteins with all metagenome-assembled genomes used in Figure 2a in Newick format.

Supplementary Dataset 13 Concatenated ribosomal protein tree of 16 ribosomal proteins for all MAGs in the Bacteroidetes phylum shown in Figure 3a in Newick format.

Supplementary Dataset 14 16S rRNA gene tree in newick format for the scaffold containing a 16S rRNA gene recovered in BACT6 MAG with near neighbours determined using SILVA.

Supplementary Dataset 15 16S rRNA gene tree in newick format for the scaffold containing a 16S rRNA gene recovered in RC9_11 MAG with near neighbours determined using SILVA.

Supplementary Dataset 16 16S rRNA gene tree in newick format for the scaffold containing a 16S rRNA gene recovered in BACT17 MAG with near neighbours determined using SILVA.

Supplementary Dataset 17 16S rRNA gene tree in newick format for the scaffold containing a 16S rRNA gene recovered in PREV31 MAG with near neighbours determined using SILVA.

Supplementary Dataset 18 16S rRNA gene tree in newick format for the scaffold containing a 16S rRNA gene recovered in BACT38 MAG with near neighbours determined using SILVA.

Supplementary Dataset 19 16S rRNA gene tree in newick format for the scaffold containing a 16S rRNA gene recovered in FIRM6 MAG with near neighbours determined using SILVA.

Supplementary Dataset 20 16S rRNA gene tree in newick format for the scaffold containing a 16S rRNA gene recovered in FIRM11 MAG with near neighbours determined using SILVA.

Supplementary Dataset 21 16S rRNA gene tree in newick format for the scaffold containing a 16S rRNA gene recovered in FIRM19 MAG with near neighbours determined using SILVA.

Supplementary Dataset 22 16S rRNA gene tree in newick format for the scaffold containing a 16S rRNA gene recovered in RUM12 MAG with near neighbours determined using SILVA.

Supplementary Dataset 23 16S rRNA gene tree in newick format for the scaffold containing a 16S rRNA gene recovered in FIRM21 MAG with near neighbours determined using SILVA.

Supplementary Dataset 24 16S rRNA gene tree in newick format for the scaffold containing a 16S rRNA gene recovered in BUTY26 MAG with near neighbours determined using SILVA

Supplementary Dataset 25 16S rRNA gene tree in newick format for scaffolds containing full or partial 16S rRNA genes recovered in Tenericutes MAGs with near neighbours determined using SILVA.

Supplementary Dataset 26 16S rRNA gene tree in newick format for scaffolds containing full or partial 16S rRNA genes recovered in Saccharibacteria (TM7) MAGs with near neighbours determined using SILVA

## Data Availability

All sequencing reads have been deposited in the Sequence Read Archive under BioProject PRJNA301235, with specific numbers listed in Supplementary Dataset 1, Supplementary Table 2. Metaproteomics data are available via ProteomeXchange with identifier PXD008667. All microbial and viral genomes are publicly available from the Joint Genome Institute under the analysis project numbers listed in Supplementary Dataset 1, Supplementary Table 1.
