# Supplementary material for: Interspecies cross-feeding orchestrates carbon degradation in the rumen ecosystem
Source: Nat Microbiol. 2018 Oct 24;3(11):1274–84. doi: 10.1038/s41564-018-0225-4 (PMC6784887; doi:10.1038/s41564-018-0225-4)
Supplement: Supplementary file 1 — Supplementary Discussion, Supplementary Figures 1–5, Supplementary References, Supplementary Dataset legends. [file 41564_2018_225_MOESM1_ESM.pdf]

nature microbiology

SUPPLEMENTARY INFORMATION

https://doi.org/10.1038/s41564-018-0225-4

In the format provided by the authors and unedited.

Interspecies cross-feeding orchestrates carbon degradation in the rumen ecosystem

Lindsey M. Solden[1], Adrian E. Naas[2], Simon Roux[1], Rebecca A. Daly[1], William B. Collins[3], Carrie D. Nicora[4], Sam O. Purvine[4], David W. Hoyt[4], Julia Schückel[5], Bodil Jørgensen[5], William Willats[6], Donald E. Spalinger[7], Jeffrey L. Firkins[8], Mary S. Lipton[4], Matthew B. Sullivan[1,9], Phillip B. Pope[2]* and Kelly C. Wrighton[1]*

[1]Department of Microbiology, The Ohio State University, Columbus, OH, USA. [2]Faculty of Chemistry, Biotechnology and Food Science, Norwegian University of Life Sciences, Aas, Norway. [3]Alaska Department of Fish and Game, Division of Wildlife Conservation, Palmer, AK, USA. [4]Pacific Northwest National Laboratory, Richland, WA, USA. [5]Department of Plant and Environmental Sciences, University of Copenhagen, Copenhagen, Denmark. [6]School of Natural and Environmental Sciences, Newcastle University, Newcastle upon Tyne, UK. [7]Department of Biology, University of Alaska Anchorage, Anchorage, AK, USA. [8]Department of Animal Sciences, The Ohio State University, Columbus, OH, USA. [9]Department of Civil, Environmental and Geodetic Engineering, The Ohio State University, Columbus, OH, USA. *e-mail: phillip.b.pope@gmail.com; kwrighton@gmail.com

Supplementary Materials for:
Interspecies cross-feedings orchestrate carbon degradation in the rumen ecosystem

Lindsey M. Solden, Adrian E. Naas, Simon Roux, Rebecca A. Daly, William B. Collins, Carrie D. Nicora, Sam O. Purvine, David W. Hoyt, Julia Schückel, Bodil Jorgensen, William Willats, Donald E. Spalinger, Jeffrey L. Firkins, Mary S. Lipton, Matthew B. Sullivan, Phillip B. Pope*, Kelly C. Wrighton*

* Co-corresponding authors
To be submitted to Nature Microbiology

This PDF file includes:

I. Supplementary Discussion
II. Supplementary Figures
III. Supplementary References

I. Supplementary Discussion

Metabolic summary and naming description for lineages first resolved here
In order to resolve the placement of genomes that have never been sampled before our dataset, we recruited near neighbors from recently published or publicly available rumen metagenomic datasets[1-5]. This included genomes from the Uncultured Bacteria and Archaea (UBA) dataset[1], 99 partial genomes from a moose metagenome (MMAG)[2], and other cattle rumen metagenomes (RUG, Hungate 1000, and Hess)[3-5]. Here, for each of our genomes belonging to previously undescribed taxonomies (e.g., representing genera, families, orders, classes) we provide: a summary of their metabolic potential, proteins detected in metaproteomics, and the proposed taxonomy using the established *Candidatus* (*Ca.*) classification for the uncultivated majority[6]. Additionally, we provide a list of genomes from the databases above, which belong to each of these proposed taxonomies (Supplementary Dataset 1, Table 3). Finally, for the phylogenetic placement of all genomes sampled, we provide the trees in newick format as Supplementary Datasets 2-26. Specific trees for named organisms are described here.

Description of Saccharibacteria, Lentisphaerae, Tenericutes, and Proteobacteria phyla taxonomies uncovered here
We recovered four Saccharibacteria genomes (TM71, TM72, TM73, TM74), all of which are the same genera within a previously unknown class in the Saccharibacteria (TM7) (Supplementary Dataset 6). Two of the three genomes contain a partial 16S rRNA gene fragment, which are monophyletic (Supplementary Dataset 26). These genomes encode the metabolic potential to ferment simple sugars into lactate or acetate (Fig. 3, Supplementary Dataset 1, Table 4), however we did not detect any of these proteins in our metaproteomics. These genomes were included in the recent update to the phylogeny of the TM7 phyla and were determined to belong to the G3 group originating from mammalian sources (unpublished data). For these genomes we propose the names *Ca.* Nanosyncoccalia (class), *Ca.* Nanosyncoccales (order), *Ca.* Nanosynecchococcus (family), *Ca.* Nanosyncoccus (genus).

We also recovered two genomes in the Lentisphaerae phylum (Supplementary Dataset 8). Metabolic reconstruction of these genomes suggested a fermentative lifestyle, with a partial EMP pathway, likely resulting in acetate production, but we did not detect these proteins in our metaproteomics. One of these genomes is similar to three previously recovered metagenomes from Rifle, CO (RifOxyA12, RifoxyGWF2, RifOxyC12)[7], and together represent an undescribed class in the Lentisphaerae. The other genome in our dataset (LENT2) is similar to UBA genomes (UBA1407, UBA1724) and one rifle genome (RifoxyB12)[7], which are part of the class Lentisphaeria, but are not classified beyond this (Supplementary Dataset 11).

All of our recovered Tenericutes genomes (TENER1-4) are monophyletic and can be assigned to the Mollicutes class (Supplementary Dataset 2). Two of these genomes contain a partial 16S rRNA gene fragment that belongs to the order called RF9, which is an order lacking a cultivated representative and is prevalent across 99% of ruminant animals sampled[8] (Supplementary Dataset 25). Based on AAI analyses, this monophyletic clade all belong to the same family[9], which we propose the name *Ca.* Tuttuvakaceae after the Inuit word for moose “Tuttuvak.” This family currently lacks sufficient genomic sampling to resolve genera. Our most dominant and active Tenericutes genome (TENER4) may rely on the fermentation of proteins and amino acids

because in proteomics we only detected hypothetical proteins, endopeptidases, and ribosomal proteins. Two of our *Ca.* Tuttuvakaceae genomes (TENER1, TENER4) encode full pathways for glycolysis, while the other two (TENER2, TENER3) only encode the ability to breakdown 3-carbon sugars (Fig. 2). All genomes encode few glycoside hydrolases indicating reliance on fermentation of glucose or glycerol to acetate, the latter product providing energy for the ruminant.

Within the Proteobacteria, we reconstructed one genome in the Aeromonadaceae family within the Gammaproteobacteria. This genome is monophyletic with 6 genomes from the RMG and MMAG databases, and likely represent a previously unsampled genus, potentially exclusive to the rumen, however these were lower quality, and did not contain a 16S rRNA gene sequence. Given the lack of confident assignment a name was not provided for this genus (Supplementary Dataset 1, Table 3), however the genome was deposited to JGI to aid in the resolution of this group in future studies.

Description of groups within the Firmicutes uncovered here

In order to confidently assign our genomic bins, we used a combination of genomic and single gene (16S rRNA) databases. Some phyla, such as the Firmicutes do not have agreement across these databases[10]. For instance, in NCBI *Catabacter* belongs to the Catabacteraceae family within the Clostridiales[11]. In Silva, this family does not exist but is encompassed in the Christensenellacae family[12]. Additionally, many named genera are not monophyletic, as genera such as *Ruminococcus*, *Clostridium*, and *Eubacterium* can be found in many separate monophyletic groups. This incongruence prevented the genus-level assignment of six of our Lachnospiraceae genome bins (FIRM21, FIRM19, FIRM17, FIRM15, FIRM16, FIRM27).

Three of our genomes (FIRM4-6) belong to the Clostridiales vadinBB60 group family within the Clostidiales Incertae Sedis order (Supplementary Dataset 3 and 19). FIRM5 and FIRM6 only contain the capacity to degrade 3-carbon sugars and were not detected in our metaproteomics. FIRM4 has the full glycolysis pathway and has the ability to produce acetate and hydrogen. These two groups are found on separate monophyletic clades, however pairwise AAI results could not distinguish separate genera as all of the genomes had <65% pairwise AAI[9]. In metaproteomics we detected proteins from FIRM4 for the degradation of mixed linked glucans indicating this groups potential importance in carbon degradation. This group was also detected in UBA and RMG datasets (Supplementary Dataset 1, Table 2).

Within the Ruminococcaceae we resolved one previously undescribed genus, with two of our genomes (FIRM7, FIRM8), and genomes from the RMG (2) and UBA (8) datasets (Supplementary Dataset 3 and 10). These genomes encode the capacity to ferment many sugars including rhamnose, xylose, fucose, and glucose fermentation. FIRM8 encodes the capacity to degrade starch, chitin, galactan, xylan, and glucans, while FIRM7 can mainly cleave hemicellulose polymers like xylan, mannan and glucan. FIRM8 is expressing genes for rhamnose fermentation, while FIRM7 contains a highly expressed (11 unique peptides) GH16 likely involved in cellulose degradation highlighting the differentiation within a genus for participation in the anaerobic carbon cycle. For this group of organisms we propose the genus name *Ca.* Vansoestibacter after the author of <u>Nutritional Ecology of the Ruminant</u> Dr. Peter J. Van Soest[12].

We also resolved a second genus with no isolated representatives in the Ruminococcaceae using two of our genomes (FIRM13 and FIRM11), which is supported by a full length (1,506 bp) 16S rRNA gene fragment in FIRM11 that is 97% similar to the {Eubacterium} coprostanoligenes group (EU844054, KC162980), with brackets indicating that the taxonomy of this group needs to be reassigned[13] (Supplementary Dataset 3 and 20). FIRM13 can utilize many sugars (glucose, fucose, arabinose, galactose and mannose) and is expressing genes for acetate production (acetate kinase). FIRM13 also contains two dockerin, and two cohesin molecules and 21 glycoside hydrolases (GH5, GH31, GH3, GH4, GH20, GH63), with one scaffold containing three GHs, including Cellulase M, indicating that this organism may use cellulosomes to degrade complex plant polymers, although no evidence for this expression was found in the metaproteomic data[14]. FIRM11 is expressing genes for fermenting fucose via glycolysis and also has the capacity to ferment galactose, xylose, mannose and rhamnose. As genomes from the moose metagenome[2] assisted in the resolution of this genus, (105 bin19, 103 bin67) we propose to name this genus *Ca.* Algibacter for the Swedish word for moose "Älg".

One of our genomes (FIRM20) belongs to a monophyletic clade with other genomes sequences as part of the Hungate 1000 project (http://www.rmgnetwork.org/hungate1000.html) (Supplementary Dataset 1, Table 3; Supplementary Dataset 3 and 11). FIRM20 encodes the capacity to ferment fucose, arabinose, galactose, xylose, and mannose, and is expressing genes for fucose fermentation. We propose to name this group of organisms within the Lachnospiraceae *Ca.* Hungatadium after Dr. Robert Hungate.

Description of sampled families within the Bacteroidales
Within the Bacteroidetes we sampled genomes from at least 10 families within the Bacteroidales, resolving three families and three genera (Supplementary Dataset 10). Notably, with the combined rumen datasets[1-5] and our genomes[15], the BS11 family and the previously described isolate *Lentimicrobium saccharophilum* within the proposed Lentimicrobiaceae form a monophyletic family, with six currently sampled monophyletic groups (Supplementary Dataset 11). Previous phylogenetic analyses of this group utilized the Greengenes database release 05_13, which did not contain BS11 as a classification[14]. The metabolic capacity of BS11 members was previously described by our group[15]. This family now contains 52 members, with three currently resolved genera, including *Lentimicrobium*[16], *Ca.* Alcium and *Ca.* Hemicellulyticus[15].

In this manuscript we resolved 3 other families within the order Bacteroidales, a group which single gene analyses have uncovered across many host ecosystems but has remained largely unresolved until recent metagenomic sampling[17]. Genome BACT8 belongs to a previously unknown family, which only contains genomes from rumen metagenomic datasets, and thus we propose the family name *Ca.* Ruminaceae (Supplementary Dataset 10 and 11). BACT8 encodes the capacity to ferment fructose and galactose, and to produce lactate and succinate. It contains one PUL system for arabinan degradation, and three other PULs for which a substrate could not be confidently determined. In metaproteomics we detected proteins involved in gliding motility, ribosomal proteins and one protein within a PUL that a substrate could not be assigned.

Our phylogenetic analyses resolved a second family in the Bacteroidales, here named *Ca.* Hungataceae (after Robert Hungate, the father of Microbiology), composed of BACT22 from our

dataset, genome AJ from Hess 2011 and two UBA genomes[1,4] (Supplementary Dataset 10 and 12). BACT22 is a highly versatile organism with the capacity to utilize every sugar examined, except rhamnose, and can degrade many different hemicellulose polymers. BACT22 is one of our hub genomes as it is expressing genes for the degradation of many different substrates (mannan, glucose, mannose, xylose, arabinose, and galactose).

The third resolved family resolved here in the Bacteroidales (proposed named *Ca.* Denaliaceae after the National park in Alaska where moose are well-studied) contains BACT18, and seven other genomes recovered from anaerobic reactors and elephant feces metagenomes (Supplementary Dataset 10). BACT18 has the capacity to degrade xyloglucan, and cleave many sugars from polymeric substrates. It also encodes the capacity to ferment all sugars examined. BACT18 also encodes respiratory capabilities with a full NADH dehydrogenase complex I and fumarate reductase. BACT18 was one of our only Bacteroidetes genomes found in the sugar utilization trophic level, as we detected proteins for the degradation of rhamnose, arabinose, and xylose but not for the degradation of plant polymers. This could be a result of the PULs in BACT18 lacking confident substrate annotation or the low abundance of BACT18 in our metagenomes.

Description of genera within known Bacteroidales families uncovered here

In addition to three families, we also resolved three genera existing within established families in the order Bacteroidales. One genus contains three genomes that clade with *Paraprevotella clara* by concatenated ribosomal protein analyses, but without significant bootstrap support (Supplementary Dataset 10). One of these genomes contains a partial 16S rRNA gene (374 base pairs) that is 90% identical to Prevotellaceae UCG-003 genus (Supplementary Dataset 18). These results suggest that these genomes are the first genomic sampling of a genus here named *Candidatus* Palmerella after Palmer, Alaska where our study took place (BACT36, BACT38, BACT39). All of these genomes encode an Rnf complex that likely participates in ion-motive electron transport[18], are lacking an NADH dehydrogenase complex I, contain genes for central glycolysis and encode the capacity for the degradation of fructose, galactose, and arabinose. There is differential capacity between these genomes to ferment rhamnose, mannose, xylose and fucose. BACT39 was the only *Ca.* Palmerella genome detected in our metaproteomics and is expressing genes for the degradation of xylan and the fermentation of xylose and mannose, indicating a role in hemicellulose breakdown.

Also within the Prevotellaceae, three of our genomes (BACT24, BACT25, BACT30) and nine UBA genomes all from rumen metagenomic datasets are monophyletic away from previously described genera within Prevotellaceae (Supplementary Dataset 10 and 11). These genomes encode a wide capacity of polymer degradation, with the exception of chitin. Additionally, these genomes contain complete glycolysis pathways, an Rnf complex, and the capacity to produce succinate and acetate. Here we propose the name *Ca.* Alyeska, after the archaic spelling of Alaska where our study took place. One *Ca.* Alyeska bin 25 is a hub genome, expressing genes for the degradation of many compounds, highlighting this groups' importance in the rumen.

The third genus we resolved was within the S24-7 (Muribaculaceae) family, with one of our genomes (BACT21) and seven genomes from the UBA, RMG and MMAG datasets (Supplementary Dataset 10 and 12). This genome encodes a wide respiratory capacity, as we

detected cytochrome b1, cytochrome d ubiquinol, NADH dehydrogenase complex I, and fumarate reductase. We did not detect the expression of this metabolism; thus we predict that BACT21 is breaking down starch, mannan, and fructan and fermenting glucose. For this genus we propose the name *Ca.* Ormerodus after the first author of the paper containing the first S24-7 genomes and the resolution of many previously undescribed families in the Bacteroidales[17].

Finally we also sampled seven genomes from the RC9 gut group genus within the Rikenellaceae family. This genus (labeled as unclassified Bacteroidales in Greengenes) contains 10,855 accession numbers in Silva obtained from rumen, cecum, feces, and wastewater samples[13]. With our genomes (7) and recently published genomes from others[1,2], this genus now contains genomes from 80 members (Supplementary Dataset 11). RC9_11 also contains a 16S rRNA gene sequence that confirming this phylogeny (Supplementary Dataset 15). Surprisingly, despite prior genomic sampling, the metabolism of these lineages remained undefined. Here we reconstructed the metabolism of our RC9 gut group genomes and identified the potential for hemicellulose and pectin polymer degradation using the Polysaccharide Utilization Loci (PULs). The RC9 gut group sp. genomes encode up to 16 PULs per genome and on average are expressing genes encoded in PULs for the degradation of three different substrates. RC9_13, RC9_15, and RC9_11 are the most active RC9 members expressing genes within PULs for the degradation of pectin, starch, glucans, mannan, peptides, and arabinans (Fig. 4). Although active in carbon polymer degradation, the RC9 gut group sp. genomes are also expressing genes for sugar fermentation. Unlike the *Prevotella* sp., the RC9 gut group sp. are expressing genes for the fermentation of one sugar and it is typically a sugar present in the polymers they can degrade. For instance, RC9 gut group sp. bin RC9_10 is expressing genes for pectin and starch degradation, and genes for the fermentation of rhamnose, a common pectin sugar. RC9 gut group sp. bin RC9_13, is expressing PUL for the degradation of arabinan, pectin, and xylan and is fermenting arabinose, rhamnose, and xylose. This metabolically versatile genus within the Rikenellaceae now has a metabolic role assigned and a functional activity that can be used for the cultivation of this genus or to infer the role of these organisms in single gene studies.

Respiratory capacity recovered in genomes
The reduction of trimethylamine-N-oxide (TMAO) to trimethylamine (TMA) has not been seen previously in the rumen before, but has been observed in other cultured organisms from the gut[18]. While we detected the presence of these genes in our genomes, we did not detect any of these metabolisms in our metaproteomics. Furthermore, we did not detect TMAO or the resulting product TMA in our winter rumen fluid NMR data. We did detect TMA in our spring and summer rumen fluids, which may indicate that this metabolism is occurring in the rumen under different dietary conditions. TMA can also result from phosphotidylcholine degradation, which is found in many plant membranes[19], and can be further oxidized to TMAO, which requires oxygen. The capacity for anaerobic respiration using fumarate has been observed in *Prevotella, Porphyromonas, Fibrobacter*, and *Ruminococcus* species isolated from the rumen, although this gene can also operate during fermentation for succinate production[20] and may be a mechanism for rumen organisms to maintain low partial H_2 pressures.

Auxiliary metabolic genes detected on viral contigs
Of the seven AMGs detected here, four are likely involved in sulfur metabolism pathways, with cysteine desulfurase (3) and phosphoadenosine phosphosulfate reductase (1) genes detected on

four viral contigs. The presence of these genes on viral contigs was previously observed in the rumen[21], marine[22], and human gut viromes[23], highlighting the importance of sulfur during viral infection across ecosystems. We also detected three viral contigs with AMGs for the synthesis of Tetrahydrobiopterin (6-pyruvoyltetrahydropterin synthase). Tetrahydrobiopterin is used as a cofactor in the synthesis of aromatic amino acids in mammals, however the role of this metabolite in bacteria is unknown[24].

II. Supplementary Figures

Supplementary Figure 1. (A) Sampling design for rumen-fistulated moose. Rumen fluid (circles) and feces (squares) were sampled from each moose prior to the seasonal diet study, during all native diets (Spring, Summer, Winter), and after the winter sampling period. Collection dates are labeled at the bottom for 2014-2015. The first week of the spring diet was also sampled to determine how quickly microbial communities change from pelleted feed resulting in a total 24-time series samples on native diets. Pelleted feed samples were taken for comparison to native diets. Within each seasonal diet, microbial communities were statistically indistinguishable between moose and between sampling dates. Sample identified in red was selected for deep metagenomic sequencing. (B) Sample for metagenomics was separated into fractions to allow for the greatest recovery of genomes. (C) Our prior manuscript[15] includes 16S rRNA gene data, dietary analyses, rumen fluid short-chain fatty acid measurements, rumen fluid fiber quantification, and 4 metagenome-assembled genomes (MAGs) recovered from metagenomic samples. (D) This manuscript details the entire metagenome, with all associated MAGs, viral contigs, metaproteomes, and metabolites. Additionally, we more directly characterized the plant polymers detected in the rumen fluid from all samples and incorporated metabolites, MAGs, and metaproteomes into a network analysis to generate predictions on functional roles of MAGs recovered in the winter rumen fluid. Finally, we confirmed these predictions using biochemical assays to generate a hypothetical model of a highly expressed PUL in metaproteomics.

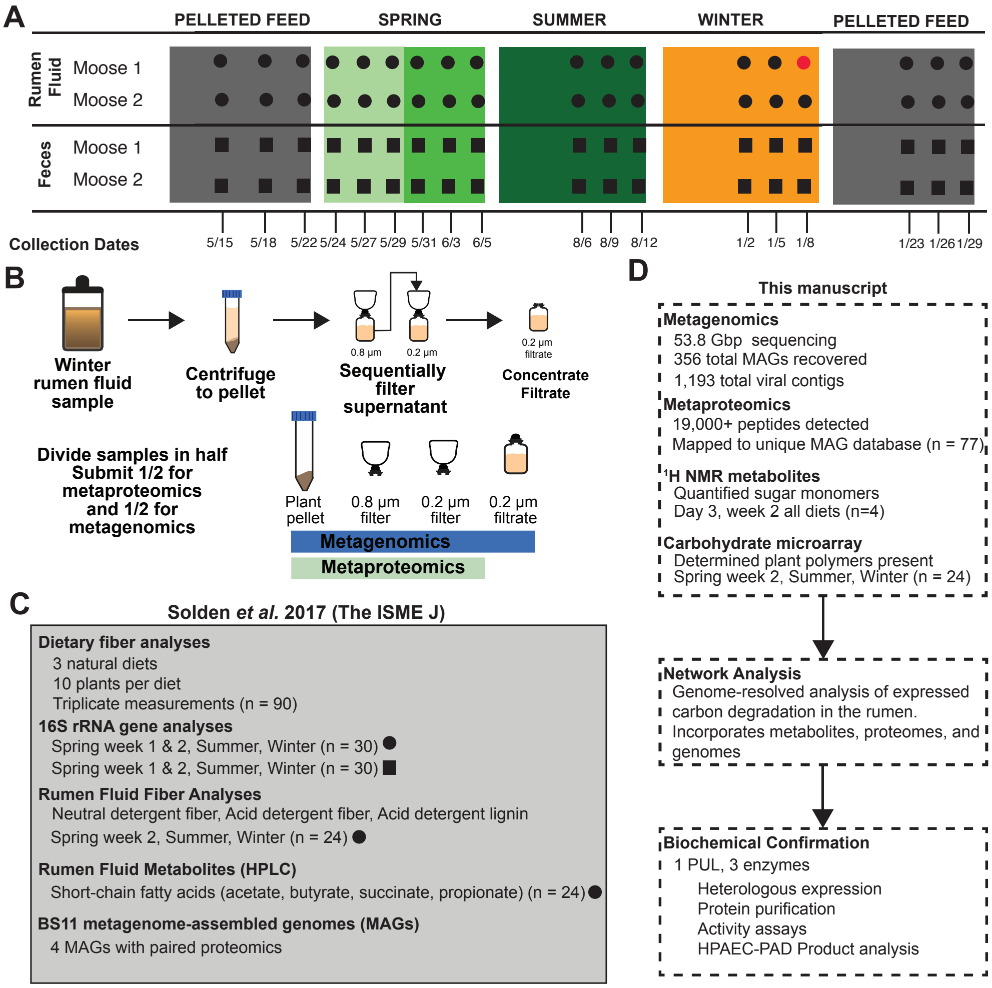

Supplementary Figure 2. Rank Abundance curve of the average abundances of taxa in the Spring (6 samples) and Winter (6 samples) previously published in Solden *et al.* 2017[15]. Overlayed are stars to highlight bars that match genomes sampled in this manuscript, including genomes previously published (yellow stars), genomes containing a 16S rRNA gene (>100 bp, red star), or genomes that did not contain a 16S rRNA gene but match enriched genera (blue star).

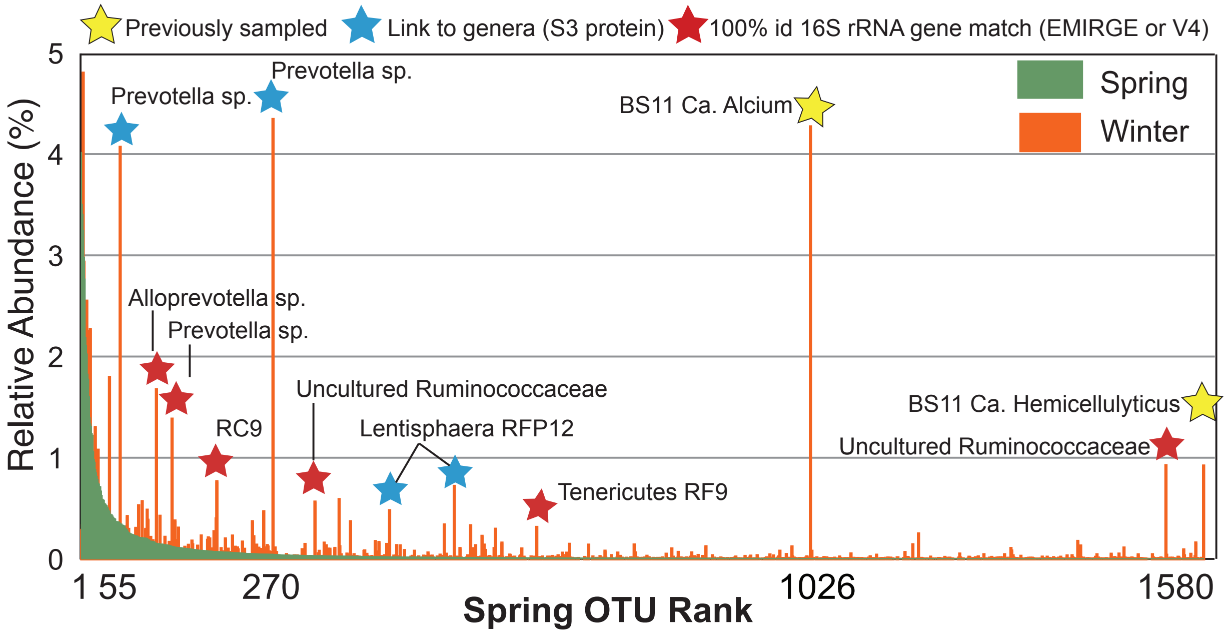

Supplementary Figure 3. Taxonomic classification and relative abundance of recovered genomes (77) across all metagenomes (4). Bars on the left axis indicate phylum (Sa, Saccharibacteria; Lent, Lentisphaera; Tener, Tenericutes; Prot, Proteobacteria; F, Fibrobacteres; E, Euryarchaeota; S, Spirochaetes). Circles represent genome bins and are colored by the highest known classification. Black bars separate family level groupings, while grey bars separate genera, within the same family. Genome names are listed on the right, with proposed names of groupings uncovered here in bold. Parentheses denote total number of genomes in this group, with asterix indicating the presence of a 16S rRNA gene sequence (>300bp) in at least one genome from this group. Genomes recovered here that contain a 16S rRNA gene fragment are noted with starred circles.

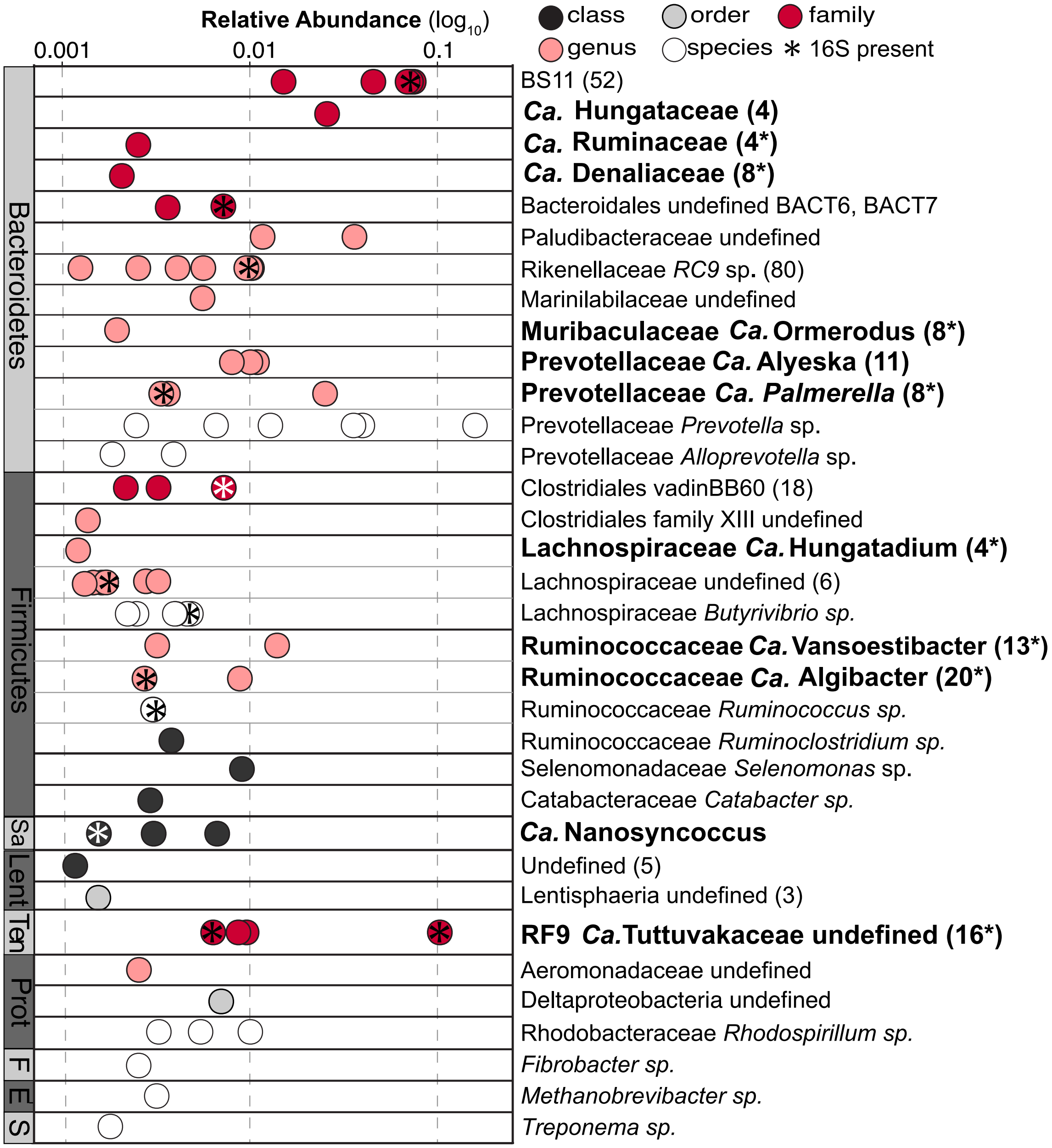

Supplementary Figure 4. Measured plant polymers in winter rumen fluid (n=6). CoMPP values (abundance) of detected cellulose (black), hemicellulose (dark grey), and pectin (light grey) polymers. Data points are represented in white circles. Box plots represent the medium, first quartile and third quartile values.

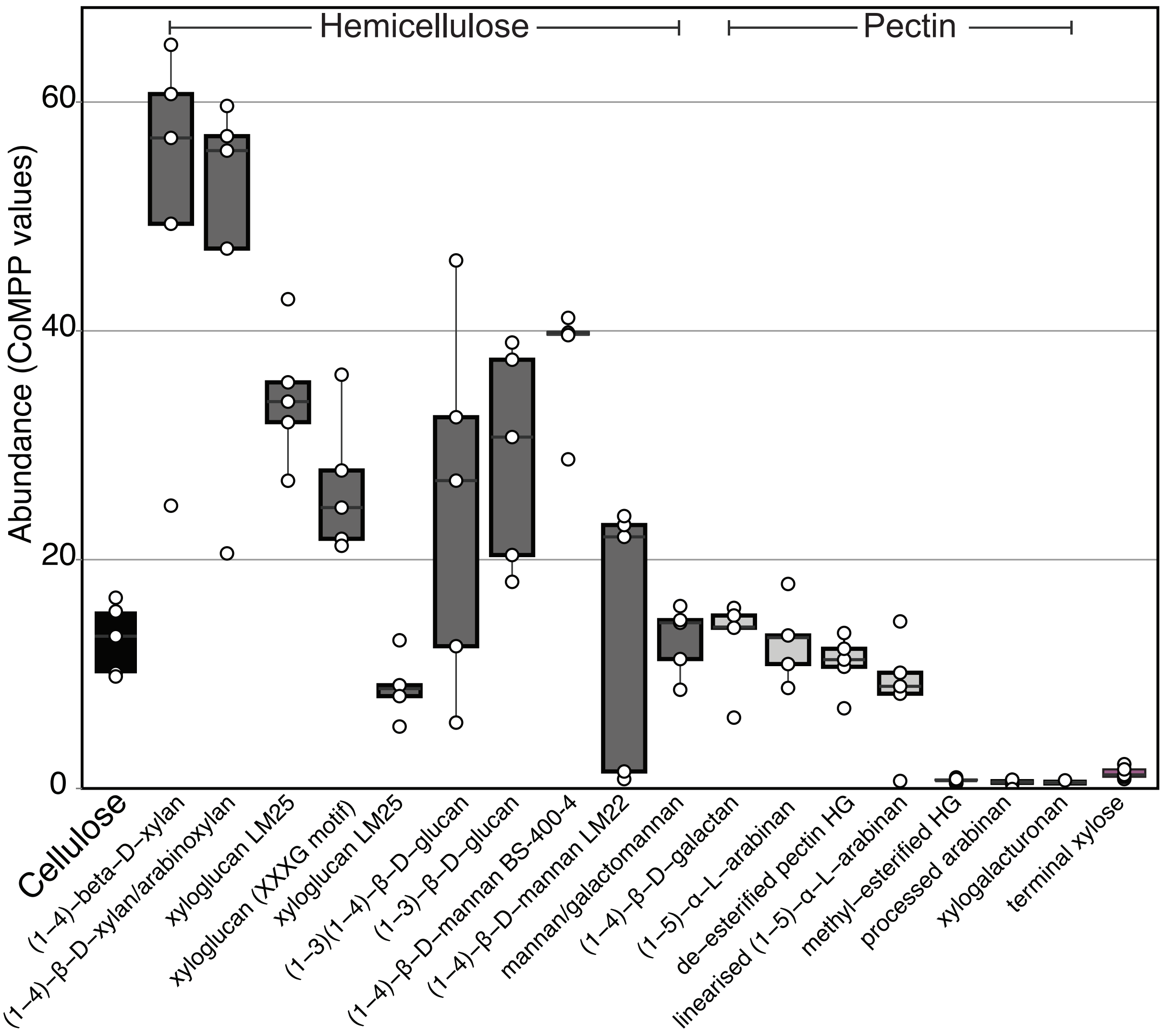

Supplementary Figure 5. Taxonomic assignment of 1,193 viral contigs and expression of viral proteins. (A) The number and affiliated taxonomy of viral genomes across metagenomes. The post-0.2 um filtrate had the most viral contigs recovered as many of the microorganisms remained on the filter. (B) Rank abundance curve of all viruses detected and their total coverage across all 4 metagenomes. Red bars indicate genomes with proteins detected in metaproteomics.

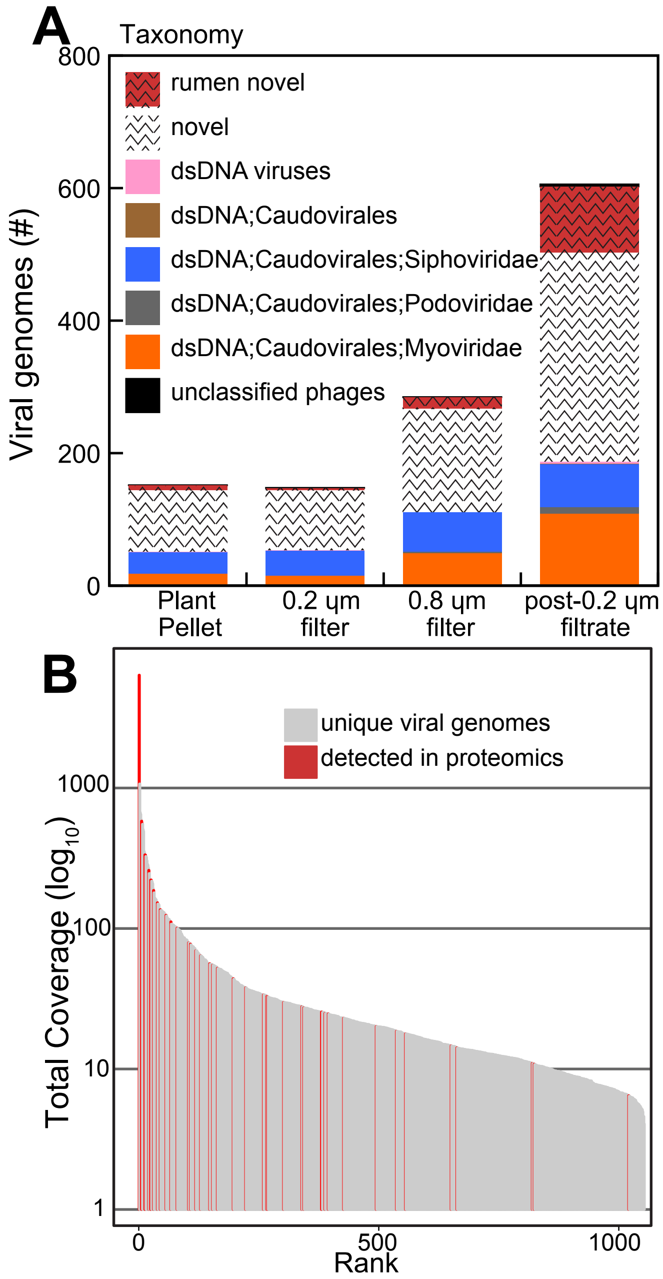

III. Supplementary References

1 Parks, D. H. *et al.* Recovery of nearly 8,000 metagenome-assembled genomes substantially expands the tree of life. *Nature microbiology* **2**, 1533 (2017).

2 Svartström, O. *et al.* Ninety-nine de novo assembled genomes from the moose (Alces alces) rumen microbiome provide new insights into microbial plant biomass degradation. *The ISME journal* (2017).

3 Stewart, R. D. *et al.* Assembly of 913 microbial genomes from metagenomic sequencing of the cow rumen. *Nature communications* **9**, 870 (2018).

4 Hess, M. *et al.* Metagenomic discovery of biomass-degrading genes and genomes from cow rumen. *Science* **331**, 463-467, doi:10.1126/science.1200387 (2011).

5 Seshadri, R. *et al.* Cultivation and sequencing of rumen microbiome members from the Hungate1000 Collection. *Nature biotechnology* (2018).

6 Konstantinidis, K. T. & Rossello-Mora, R. Classifying the uncultivated microbial majority: A place for metagenomic data in the Candidatus proposal. *Syst Appl Microbiol* **38**, 223-230, doi:10.1016/j.syapm.2015.01.001 (2015).

7 Anantharaman, K. *et al.* Thousands of microbial genomes shed light on interconnected biogeochemical processes in an aquifer system. *Nature communications* **7** (2016).

8 Henderson, G. *et al.* Rumen microbial community composition varies with diet and host, but a core microbiome is found across a wide geographical range. *Sci Rep* **5**, 14567, doi:10.1038/srep14567 (2015).

9 Konstantinidis, K. T., Rossello-Mora, R. & Amann, R. Uncultivated microbes in need of their own taxonomy. *ISME J*, doi:10.1038/ismej.2017.113 (2017).

10 Yutin, N. & Galperin, M. Y. A genomic update on clostridial phylogeny: Gram‐negative spore formers and other misplaced clostridia. *Environmental microbiology* **15**, 2631-2641 (2013).

11 Pruitt, K. D., Tatusova, T., Brown, G. R. & Maglott, D. R. NCBI Reference Sequences (RefSeq): current status, new features and genome annotation policy. *Nucleic acids research* **40**, D130-D135 (2011).

12 Van Soest, P. *Nutritional Ecology of the Ruminant*. (1994).

13 Quast, C. *et al.* The SILVA ribosomal RNA gene database project: improved data processing and web-based tools. *Nucleic Acids Res* **41**, D590-596, doi:10.1093/nar/gks1219 (2013).

14 Schwarz, W. The cellulosome and cellulose degradation by anaerobic bacteria. *Applied microbiology and biotechnology* **56**, 634-649 (2001).

15 Solden, L. M. *et al.* New roles in hemicellulosic sugar fermentation for the uncultivated Bacteroidetes family BS11. *The ISME journal* **11**, 691-703 (2017).

16 Sun, L. *et al.* Lentimicrobium saccharophilum gen. nov., sp. nov., a strictly anaerobic bacterium representing a new family in the phylum Bacteroidetes, and proposal of Lentimicrobiaceae fam. nov. *International journal of systematic and evolutionary microbiology* **66**, 2635-2642 (2016).

17 Ormerod, K. L. *et al.* Genomic characterization of the uncultured Bacteroidales family S24-7 inhabiting the guts of homeothermic animals. *Microbiome* **4**, 36 (2016).

18 Biegel, E. & Muller, V. Bacterial Na+-translocating ferredoxin:NAD+ oxidoreductase. *Proc Natl Acad Sci U S A* **107**, 18138-18142, doi:10.1073/pnas.1010318107 (2010).

19 Neill, A. R., Grime, D. W. & Dawson, R. Conversion of choline methyl groups through trimethylamine into methane in the rumen. *Biochemical Journal* **170**, 529-535 (1978).
20 Asanuma, N. & Hino, T. Activity and properties of fumarate reductase in ruminal bacteria. *The Journal of general and applied microbiology* **46**, 119-125 (2000).
21 Anderson, C. L., Sullivan, M. B. & Fernando, S. C. Dietary energy drives the dynamic response of bovine rumen viral communities. *Microbiome* **5**, 155 (2017).
22 Sharon, I. *et al.* Comparative metagenomics of microbial traits within oceanic viral communities. *The ISME journal* **5**, 1178 (2011).
23 Reyes, A. *et al.* Viruses in the faecal microbiota of monozygotic twins and their mothers. *Nature* **466**, 334 (2010).
24 Kong, J. S. *et al.* 6-Pyruvoyltetrahydropterin synthase orthologs of either a single or dual domain structure are responsible for tetrahydrobiopterin synthesis in bacteria. *FEBS letters* **580**, 4900-4904 (2006).

Supplementary Guide

Supplementary Dataset 1. Excel file with all supplementary tables.

Supplementary Table 1. Taxonomic summary, genome completion, and quality of all unique, near-complete genomes.

Supplementary Table 2. Assembly statistics and SRA numbers for metagenomes.

Supplementary Table 3. Genomes from other datasets that belong to taxonomies resolved here. Abbreviations of datasets are as follows: UBA, Uncultured Bacteria and Archaea2; RMG, Rumen Meta-Genome3; MMAG, Moose Metagenome-Assembled Genomes4.

Supplementary Table 4. Genes mined for metabolic reconstruction of genomic potential in genome bins.

Supplementary Table 5. All polysaccharide utilization loci recovered with a detailed list of glycoside hydrolases surrounding SusCD-like genes. All predicted substrates were compared to previously published PUL systems25-33.

Supplementary Table 6. Full annotation of all viral scaffolds with proteins detected in metaproteomics.

Supplementary Table 7. Carbon polymer abbreviations used in Figure 5 with corresponding CoMPP probe.

Supplementary Dataset 2. Concatenated ribosomal protein tree of 16 ribosomal proteins for all MAGs in the Tenericutes phylum, with reference sequences in newick format.

Supplementary Dataset 3. Concatenated ribosomal protein tree of 16 ribosomal proteins for all MAGs in the Firmicutes phylum, with reference sequences in newick format.

Supplementary Dataset 4. Concatenated ribosomal protein tree of 16 ribosomal proteins for all MAGs in the Spirochaetes phylum, with reference sequences in newick format.

Supplementary Dataset 5. Concatenated ribosomal protein tree of 16 ribosomal proteins for all MAGs in the Fibrobacteres phylum, with reference sequences in newick format.

Supplementary Dataset 6. Concatenated ribosomal protein tree of 16 ribosomal proteins for all MAGs in the Saccharibacteria (TM7) phylum, with reference sequences in newick format.

Supplementary Dataset 7. Concatenated ribosomal protein tree of 16 ribosomal proteins for all MAGs in the Euryarchaeota phylum, with reference sequences in newick format.

Supplementary Dataset 8. Concatenated ribosomal protein tree of 16 ribosomal proteins for all MAGs in the Lentisphaera phylum, with reference sequences in newick format.

Supplementary Dataset 9. Concatenated ribosomal protein tree of 16 ribosomal proteins for all MAGs in the Proteobacteria phylum, with reference sequences in newick format.

Supplementary Dataset 10. Concatenated ribosomal protein tree of 16 ribosomal proteins for all MAGs in the Bacteroidetes phylum, with reference sequences in newick format.

Supplementary Dataset 11. Full ribosomal protein S3 tree in newick format shown in Figure 1.

Supplementary Dataset 12. Full concatenated ribosomal protein tree of 16 ribosomal proteins with all metagenome-assembled genomes used in Figure 2A in newick format.

Supplementary Dataset 13. Concatenated ribosomal protein tree of 16 ribosomal proteins for all MAGs in the Bacteroidetes phylum shown in Figure 3A in newick format.

Supplementary Dataset 14. 16S rRNA gene tree in newick format for scaffold recovered in BACT6 MAG with near neighbors determined using SILVA.

Supplementary Dataset 15. 16S rRNA gene tree in newick format for scaffold recovered in BACT11 MAG with near neighbors determined using SILVA.

Supplementary Dataset 16. 16S rRNA gene tree in newick format for scaffold recovered in BACT17 MAG with near neighbors determined using SILVA.

Supplementary Dataset 17. 16S rRNA gene tree in newick format for scaffold recovered in PREV31 MAG with near neighbors determined using SILVA.

Supplementary Dataset 18. 16S rRNA gene tree in newick format for scaffold recovered in BACT38 MAG with near neighbors determined using SILVA.

Supplementary Dataset 19. 16S rRNA gene tree in newick format for scaffold recovered in FIRM6 MAG with near neighbors determined using SILVA.

Supplementary Dataset 20. 16S rRNA gene tree in newick format for scaffold recovered in FIRM11 MAG with near neighbors determined using SILVA.

Supplementary Dataset 21. 16S rRNA gene tree in newick format for scaffold recovered in FIRM19 MAG with near neighbors determined using SILVA.

Supplementary Dataset 22. 16S rRNA gene tree in newick format for scaffold recovered in RUM12 MAG with near neighbors determined using SILVA.

Supplementary Dataset 23. 16S rRNA gene tree in newick format for scaffold recovered in FIRM21 MAG with near neighbors determined using SILVA.

Supplementary Dataset 24. 16S rRNA gene tree in newick format for scaffold recovered in BUTY26 MAG with near neighbors determined using SILVA.

Supplementary Dataset 25. 16S rRNA gene tree in newick format for scaffold recovered in Tenericutes MAGs with near neighbors determined using SILVA.

Supplementary Dataset 26. 16S rRNA gene tree in newick format for scaffold recovered in Saccharibacteria (TM7) MAGs with near neighbors determined using SILVA.
