# Supplementary material for: Interspecies cross-feeding orchestrates carbon degradation in the rumen ecosystem
Source: Nat Microbiol. 2018 Oct 24;3(11):1274–84. doi: 10.1038/s41564-018-0225-4 (PMC6784887; doi:10.1038/s41564-018-0225-4)
Supplement: Supplementary file 2 — Reporting Summary [file 41564_2018_225_MOESM2_ESM.pdf]

nature research

Corresponding author(s): Kelly C. Wrighton

☐ Initial submission ☐ Revised version ☒ Final submission

Life Sciences Reporting Summary

Nature Research wishes to improve the reproducibility of the work that we publish. This form is intended for publication with all accepted life science papers and provides structure for consistency and transparency in reporting. Every life science submission will use this form; some list items might not apply to an individual manuscript, but all fields must be completed for clarity.

For further information on the points included in this form, see Reporting Life Sciences Research. For further information on Nature Research policies, including our data availability policy, see Authors & Referees and the Editorial Policy Checklist.

Experimental design

1. Sample size

Describe how sample size was determined.

To our knowledge, the moose used in this study are the only two rumen fistulated moose in the world. This surgical procedure provided unparalleled access to rumen fluid samples from live moose as they were consuming and digesting food. These moose were monitored over the course of a year, as they foraged naturally three seasonal diets (spring, summer, fall/winter) in Alaska and a control pellet diet. After a one-week diet adjustment period each moose was sampled three times on the diet over a week period. Microbial community analyses using 16S rRNA gene sequencing determined that sample replicates per diet treatment were statistically similar and independent of host. These findings were published previously in The ISME Journal (Solden et al. 2017). From these prior analyses, we demonstrated that uncultivated Bacteroidetes were enriched in the winter rumen fluids. To uncover the physiological roles and substrate preferences for these uncultivated Bacteroidetes prevalent in the winter rumen fluid, here we deeply sequenced 4 metagenomes (total 53.8 Gbp) and conducted 3 metaproteomes (over 16,000 peptides) from one moose winter rumen fluid sample. This approach allowed us to sequence a single sample deeply to recover genomes from low abundant, but novel or perhaps functionally important members. The sequencing depth allocated to a single sample is much larger than most recent rumen sequencing projects to date (e.g. Brulc et al 2009, Wallace et al. 2015, Lopes et al. 2015, Svartstrom et al. 2017), offering the capacity to recover high quality genomes that may be low abundance. This approach was critical, as our goal was to create a genome resolved database for mapping the meta-proteome data to, enabling discovery of a carbon degradation food web (incorporating genome, enzyme, metabolite insights) prevalent in the rumen. These data were analyzed in combination with metabolite analyses (via carbohydrate microarrays and 1H NMR) and bichemical experiments (using enzymatic assays and HPAEC-PAD) for in-depth characterization of carbon active enzymes within polysaccharide utilization loci.

2. Data exclusions

Describe any data exclusions.

No data were excluded from the analyses

3. Replication

Describe whether the experimental findings were reliably reproduced.

16S rRNA gene data from our previous study showed that rumen fluid samples from moose consuming a winter diet were statistically indistinguishable and were considered replicates (n=6). Many of the genomes recovered here were clearly linked to 16S rRNA data collected by our team previously, demonstrating reproducibility across microbiome methods and linkages to our prior study (Solden et al., 2017). Additionally, many of the genomes recovered in this study were separately assembled and binned across samples, demonstrating methodological reproducibility. These identical or nearly identical genomes were de-replicated to identify a best representative genotype for the proteome database.

CoMPP analyses were repeated in triplicate and all attempts at replication were successful.

Enzymatic assays and HPAEC-PAD experiment were performed in triplicate, with

reliable reproduction of results.

4. Randomization

Describe how samples/organisms/participants were allocated into experimental groups.

This study examined the moose rumen microbiome on a single diet. There was only one treatment group examined in this manuscript.

5. Blinding

Describe whether the investigators were blinded to group allocation during data collection and/or analysis.

Blinding was not relevant to our study.

Note: all studies involving animals and/or human research participants must disclose whether blinding and randomization were used.

6. Statistical parameters

For all figures and tables that use statistical methods, confirm that the following items are present in relevant figure legends (or in the Methods section if additional space is needed).

n/a	Confirmed	
☐	☒	The exact sample size (n) for each experimental group/condition, given as a discrete number and unit of measurement (animals, litters, cultures, etc.)
☐	☒	A description of how samples were collected, noting whether measurements were taken from distinct samples or whether the same sample was measured repeatedly
☐	☒	A statement indicating how many times each experiment was replicated
☒	☐	The statistical test(s) used and whether they are one- or two-sided (note: only common tests should be described solely by name; more complex techniques should be described in the Methods section)
☒	☐	A description of any assumptions or corrections, such as an adjustment for multiple comparisons
☒	☐	The test results (e.g. P values) given as exact values whenever possible and with confidence intervals noted
☒	☐	A clear description of statistics including central tendency (e.g. median, mean) and variation (e.g. standard deviation, interquartile range)
☒	☐	Clearly defined error bars

See the web collection on statistics for biologists for further resources and guidance.

Software

Policy information about availability of computer code

7. Software

Describe the software used to analyze the data in this study.

All code, software descriptions, and databases used in this manuscript are described on GitHub found at https://github.com/TheWrightonLab/. Specific analyses are listed as separate repositories and are cited within the methods section as needed.

For manuscripts utilizing custom algorithms or software that are central to the paper but not yet described in the published literature, software must be made available to editors and reviewers upon request. We strongly encourage code deposition in a community repository (e.g. GitHub). *Nature Methods* guidance for providing algorithms and software for publication provides further information on this topic.

Materials and reagents

Policy information about availability of materials

8. Materials availability

Indicate whether there are restrictions on availability of unique materials or if these materials are only available for distribution by a for-profit company.

No unique materials were used

9. Antibodies

Describe the antibodies used and how they were validated for use in the system under study (i.e. assay and species).

No antibodies were used

10. Eukaryotic cell lines

a. State the source of each eukaryotic cell line used.	No eukaryotic cell lines were used
b. Describe the method of cell line authentication used.	No eukaryotic cell lines were used
c. Report whether the cell lines were tested for mycoplasma contamination.	No eukaryotic cell lines were used
d. If any of the cell lines used are listed in the database of commonly misidentified cell lines maintained by ICLAC, provide a scientific rationale for their use.	No eukaryotic cell lines were used

Animals and human research participants

Policy information about studies involving animals; when reporting animal research, follow the ARRIVE guidelines

11. Description of research animals

Provide details on animals and/or animal-derived materials used in the study.	Two female moose (Alces alces gigas), both age 12 were observed in a native pasture in Alaska during three seasons.

Policy information about studies involving human research participants

12. Description of human research participants

Describe the covariate-relevant population characteristics of the human research participants.	The study did not involve human research participants.
